# Impact of the lipid–inflammation axis on endometriosis risk: a multicenter case–control study using mediation analysis

**DOI:** 10.3389/fendo.2025.1661264

**Published:** 2025-11-13

**Authors:** Yanan Duan, Fanmao Kong, Zhaoxia Ding, Yiqing Peng, Aiping Chen, Yushuang Yao

**Affiliations:** 1 Department of Gynecology, The Affiliated Hospital of Qingdao University, Qingdao, Shandong, China; 2 Qingdao Medical College, Qingdao University, Qingdao, Shandong, China; 3 Zhongshan Road Community Health Service Center of Shinan District, Qingdao, Shandong, China, China; 4 Department of Obstetrics and Gynecology, The Affiliated Hospital of Jining Medical University, Jining, Shandong, China

**Keywords:** endometriosis, dyslipidemia, systemic immune-inflammation index, mediation analysis, case-control study

## Abstract

**Background:**

Endometriosis (EM) is often accompanied by dyslipidemia, but the causal relationship between dyslipidemia and inflammation remains unclear. This study aimed to explore the association between the lipid-inflammation axis and EM risk and to quantify the mediating role of the systemic immune-inflammation index (SII).

**Methods:**

A total of 357 EM cases and 3134 controls were included. Blood lipids and SII were assessed using logistic regression, generalized additive models (GAM), and bootstrap mediation analysis. Least absolute shrinkage and selection operator (LASSO) modeling was applied, and the model was further evaluated in an external cohort.

**Results:**

For each 1 mmol/L decrease in high-density lipoprotein cholesterol (HDL-C), EM risk increased by 55%. Conversely, each 1 mmol/L increase in triglycerides (TG) and each one-unit increase in the non-HDL-cholesterol to HDL-cholesterol ratio (NHHR) were associated with 21% and 54% higher risk, respectively. Mediation analysis suggested that 88-103% of the effects of HDL-C, TG, and NHHR on EM were mediated through SII. A nomogram incorporating these variables achieved an external validation area under the curve (AUC) of 0.93, indicating strong statistical discrimination.

**Conclusion:**

Dyslipidemia may contribute to the development of EM through systemic inflammation, with SII serving as a potential intermediate marker. These findings suggest the potential value of integrating lipid regulation and anti-inflammatory strategies for EM prevention, although further clinical validation is warranted.

## Background

Endometriosis (EM) influences roughly 10% of women in their reproductive years worldwide and stands as a leading source of persistent pelvic pain and infertility (1). Despite its substantial prevalence and clinical burden, the biological mechanisms underlying EM remain only partly elucidated, and disruptions in metabolic as well as immune regulation have attracted increasing scientific interest (2). Lipid disturbance are commonly documented among individuals with EM and correlate with more severe disease phenotypes and faster progression (3–5). Although dyslipidemia is a well-recognized cardiovascular risk factor, its direct causal relationship with cardiovascular disease in women with EM has not been firmly established, and current evidence remains largely observational (6). However, most existing studies stop at association, leaving the mechanistic connection between dysregulated lipid metabolism and ectopic-lesion formation via inflammatory pathways largely undefined.

It has been hypothesized that abnormal lipid concentrations could foster a chronic, low-grade systemic inflammatory milieu that may facilitate the extra-uterine implantation of endometrial tissue and sustain a pro-inflammatory microenvironment (7–9). In this framework, inflammation may potentially act as a pivotal mediator linking dyslipidemia to EM pathogenesis, but this remains to be empirically confirmed (4). Nevertheless, only limited research has applied formal mediation models, so the magnitude of this indirect effect remains unclear. In this context, we focused on high-density lipoprotein cholesterol (HDL-C), triglycerides (TG), and the non–HDL-cholesterol to HDL-cholesterol ratio (NHHR) as lipid exposures, as these parameters have been consistently linked to metabolic and inflammatory dysregulation and are routinely assessed in clinical settings (10). HDL-C and TG are established components of lipid metabolism with well-recognized roles in systemic inflammation, while NHHR integrates both atherogenic and anti-atherogenic fractions and may better capture the net pro-inflammatory lipid milieu. For inflammatory status, we selected the systemic immune–inflammation index (SII), calculated from platelet, neutrophil, and lymphocyte counts, as it reflects the balance between innate and adaptive immune responses and has shown prognostic relevance across diverse inflammatory and gynecologic disorders (11, 12).

To close this knowledge gap, we designed a large case-control study that concurrently measured comprehensive lipid and the systemic immune-inflammation index (SII), then employed mediation analysis to interrogate the putative bridging role of SII in the lipid-EM relationship. Mediation analysis offers a statistical framework to decompose total effects into direct and indirect pathways, thereby quantifying the proportion of EM risk potentially attributable to inflammatory mediation. However, as an observational method, it is sensitive to unmeasured confounding and cannot by itself establish definitive causality. By clarifying the “lipid-inflammation-EM” axis, our work aims to pinpoint actionable metabolic-inflammatory targets for high-risk women and provide mechanistic evidence to inform the evaluation of individualized risk-prediction frameworks.

## Methods

### Study population and design

Patient records for this retrospective case–control investigation were retrieved from the Affiliated Hospital of Qingdao University for the period January 2021–December 2024. Women newly diagnosed with EM, covering all anatomical subtypes (ovarian, peritoneal, and deep infiltrating), were included, and all cases were pathologically confirmed according to Pathophysiology, diagnosis, and management of endometriosis (2). For every case, nine female in-patients without EM who were hospitalized in the same calendar window were randomly selected as controls and frequency-matched by admission date to reduce potential time-related confounding. Controls were female in-patients admitted during the same period for benign gynecologic conditions such as uterine fibroids, benign ovarian cysts, or cervical intraepithelial lesions. Patients admitted for acute infections, active autoimmune disorders, metabolic crises, or other systemic inflammatory conditions were excluded to avoid bias in lipid and inflammatory indices. This strategy ensured both comparability between cases and controls in terms of hospital-based recruitment and a control-to-case ratio that approximated the 10% prevalence of EM in women of reproductive age in the source population.

The study adhered to the Declaration of Helsinki and was approved by the institutional ethics committee. Exclusions applied to individuals who (i) were under 18 years of age, (ii) lacked a definitive diagnosis, (iii) had any prior record of EM, (iv) had used antibiotics or lipid-altering medication continuously within the previous three months, or (v) had incomplete clinical or laboratory information. After screening, 357 incident EM cases and 3,134 controls admitted during the same period were available for analysis. Detailed anatomical classification of endometriosis (ovarian, peritoneal, or deep infiltrating) was not consistently available for all cases, as operative notes and intraoperative findings were incomplete in part of the records. Therefore, subtype-specific analyses were not performed to avoid potential misclassification bias. For laboratory parameters, all lipid and inflammatory indices were measured in the hospital’s central laboratory using standardized automated assays subject to daily internal and external quality control. The analytical precision for these assays, expressed as the standard error (SE), was obtained from institutional quality-control reports and is provided in the **Table 1** footnote. We note that between-group differences smaller than the SE should be interpreted with caution as they may fall within the expected measurement variability. For external validation, identical inclusion and exclusion criteria were applied to data from the Affiliated Hospital of Jining Medical University spanning January 2016-December 2024, yielding an independent cohort of 43 newly diagnosed EM cases and 497 controls without EM (Approval No.: 2022C064).

**Table 1:** Baseline characteristics description of study population.

	Total(n=3,491) Mean ± SD/N(%)	No-EM(n=3,134) Mean ± SD/N(%)	EM(n=357) Mean ± SD/N(%)	P value
Age, years	41.99 ± 9.33	42.08 ± 9.32	41.15 ± 9.44	0.074
Height, cm	161.25 ± 4.72	161.37 ± 4.74	160.19 ± 4.39	<0.001
Weight, kg	62.40 ± 7.29	62.40 ± 7.55	62.45 ± 4.29	0.894
BMI, kg/m^2^	24.02 ± 2.81	23.98 ± 2.87	24.39 ± 2.18	0.009
Overweight, n (%)				0.007
No	1734 (49.67%)	1581 (50.45%)	153 (42.86%)	
Yes	1757 (50.33%)	1553 (49.55%)	204 (57.14%)	
History of cardiovascular and cerebrovascular diseases, n (%)				0.030
No	3309 (94.79%)	2962 (94.51%)	347 (97.20%)	
Yes	182 (5.21%)	172 (5.49%)	10 (2.80%)	
History of diabetes, n (%)				0.192
No	3325 (95.24%)	2980 (95.09%)	345 (96.64%)	
Yes	166 (4.76%)	154 (4.91%)	12 (3.36%)	
History of cancer, n (%)				0.185
No	3445 (98.68%)	3090 (98.60%)	355 (99.44%)	
Yes	46 (1.32%)	44 (1.40%)	2 (0.56%)	
History of open surgery, n (%)				0.901
No	2240 (64.16%)	2012 (64.20%)	228 (63.87%)	
Yes	1251 (35.84%)	1122 (35.80%)	129 (36.13%)	
History of hysteroscopy and laparoscopic surgery, n (%)				0.737
No	2526 (72.36%)	2265 (72.27%)	261 (73.11%)	
Yes	965 (27.64%)	869 (27.73%)	96 (26.89%)	
Age at menarche, years	13.92 ± 1.35	13.92 ± 1.35	13.95 ± 1.38	0.664
Menstrual regularity, n (%)				<0.001
Erratical	808 (23.15%)	754 (24.06%)	54 (15.13%)	
Rule	2683 (76.85%)	2380 (75.94%)	303 (84.87%)	
Amount of menses, n (%)				0.012
Less	131 (3.75%)	119 (3.80%)	12 (3.36%)	
Normal	2983 (85.45%)	2693 (85.93%)	290 (81.23%)	
More	377 (10.80%)	322 (10.27%)	55 (15.41%)	
Dysmenorrhea, n (%)				<0.001
No	2986 (85.53%)	2830 (90.30%)	156 (43.70%)	
Yes	505 (14.47%)	304 (9.70%)	201 (56.30%)	
HLD, mmol/L	1.56 ± 0.33	1.57 ± 0.33	1.48 ± 0.32	<0.001
LDL, mmol/L	2.74 ± 0.69	2.74 ± 0.69	2.76 ± 0.70	0.501
VLDL, mmol/L	0.55 ± 0.20	0.55 ± 0.20	0.58 ± 0.23	0.024
TC, mmol/L	4.85 ± 0.88	4.86 ± 0.88	4.81 ± 0.83	0.364
TG, mmol/L	0.93 ± 0.61	0.92 ± 0.60	1.02 ± 0.68	0.007
NHHR	2.19 ± 0.66	2.17 ± 0.64	2.39 ± 0.82	<0.001
LC, 10^9^/L	1.67 ± 0.51	1.65 ± 0.50	1.87 ± 0.54	<0.001
NC, 10^9^/L	2.68 ± 0.80	2.61 ± 0.72	3.32 ± 1.08	<0.001
PLT, 10^9^/L	215.49 ± 50.58	211.29 ± 47.68	252.38 ± 59.59	<0.001
PLR	136.96 ± 39.95	136.48 ± 40.23	141.09 ± 37.24	0.039
NLR	1.67 ± 0.46	1.66 ± 0.45	1.84 ± 0.56	<0.001
SII	348.84 ± 87.35	337.16 ± 70.16	451.34 ± 140.48	<0.001

### Variables

The outcome variable in this study was EM. Covariates included age, height, weight, body mass index (BMI), overweight status, medical history (history of cardiovascular or cerebrovascular disease, diabetes, and cancer), surgical history (history of open surgery, hysteroscopy and laparoscopic surgery), and menstrual characteristics (age at menarche, menstrual regularity, amount of menses, and dysmenorrhea). Age, height, weight, BMI, overweight status, medical history, surgical history, and menstrual characteristics were obtained from routine admission interviews conducted by trained personnel. Overweight status, medical history, surgical history, cycle regularity, menstrual flow, and dysmenorrhea were treated as categorical variables. Platelet count (PLT, 10^9^/L), lymphocyte count (LC, 10^9^/L), neutrophil count (NC, 10^9^/L), platelet-to-lymphocyte ratio (PLR), neutrophil-to-lymphocyte ratio (NLR), SII, high-density lipoprotein (HDL, mmol/L), low-density lipoprotein (LDL, mmol/L), very-low-density lipoprotein (VLDL, mmol/L), total cholesterol (TC, mmol/L), triglycerides (TG, mmol/L), and NHHR were measured from fasting blood samples drawn before any treatment and delivered to the laboratory within 1 hour for analysis.

The relevant indices were calculated as follows:


$$BMI=Weight\left(kg\right)/Height{\left(cm\right)}^{2}$$

$$PLR=PLT\left(10^{9}/L\right)/LC\left(10^{9}/L\right)$$

$$NLR=NC\left(10^{9}/L\right)/LC\left(10^{9}/L\right)$$

$$SII=PLT\left(10^{9}/L\right)*NC\left(10^{9}/L\right)/LC\left(10^{9}/L\right)$$

NHHR=no−HLD(mmol/L)/HLD(mmol/L)

Selection of lipid indices, inflammatory markers, and covariates was based on prior literature and clinical relevance, ensuring inclusion of parameters with established or hypothesized links to EM pathophysiology.

### Statistical analysis

The choice of statistical models was guided by the nature of the outcome variable, the study design, and the clinical interpretability of results. Binary logistic regression was chosen as the primary modelling framework given the binary nature of the EM outcome and its wide applicability in clinical epidemiology. Generalized additive models (GAMs) and segmented regression were used to flexibly detect and quantify potential non-linear associations. Alternative modelling strategies, such as random forest or gradient boosting, were explored in preliminary analyses; although these methods achieved similar discrimination, they were excluded from the main analysis due to lower interpretability and the lack of readily translatable effect estimates for clinical use. Continuous data were summarized as means ± standard deviations, whereas categorical characteristics were described with frequencies and percentages. To identify factors associated with EM, we first carried out univariate screening and subsequently entered the candidate variables into multivariable logistic‐regression models that provided β-coefficients, P-values, odds ratios (ORs), and 95% confidence intervals (CIs).

Continuous predictors were initially modelled as continuous variables to maximize statistical power and avoid the information loss associated with categorization (13). Logistic regression assumes a linear relationship between continuous predictors and the log-odds of the outcome; violation of this assumption may bias effect estimates (13). Therefore, we assessed potential non-linear relationships using generalized additive models (GAMs), which allow flexible, data-driven estimation of exposure–response curves without pre-specifying functional forms. When the GAM-derived curves suggested threshold effects, segmented (two-piecewise) linear regression was chosen for its ability to provide interpretable inflection points that may align with clinically relevant cut-offs (14).

Possible departures from linearity between continuous predictors and EM risk were investigated with generalized additive models (GAMs), which supplied smoothed exposure-response curves. When curve shapes suggested thresholds, segmented (two-piecewise) linear regressions were fitted; break-points were determined by maximum-likelihood and evaluated with likelihood-ratio tests.

To gauge how lipid profiles might influence inflammatory status, we constructed multivariable linear models with individual lipid indices (HDL, LDL, VLDL, TG, TC, NHHR) as exposures and inflammatory markers (PLT, LC, NC, PLR, NLR, SII) as outcomes, adjusting for covariates. Building on these results, mediation analysis was performed with the mediation package in R, treating SII as the mediator linking each lipid metric to EM. Total, direct, and indirect effects were estimated, and 5–000 non-parametric bootstrap resamples produced confidence intervals. A mediation effect was declared when the indirect pathway, the total effect, and the proportion mediated were all significant and positive (15).

The overall dataset was split at random into a training cohort (70%) and an internal validation cohort (30%). Variable selection in the training set used the least absolute shrinkage and selection operator (LASSO), which applies an L1 penalty (λ) to shrink uninformative coefficients to zero and retain the most relevant predictors (16, 17). Selected variables were combined in a multivariable model that formed the basis of a clinical prediction nomogram. Model performance was appraised with receiver operating characteristic (ROC) curves, calibration plots, and decision-curve analysis (DCA) in the training data, and then confirmed in the validation data.

Robustness across patient subgroups was checked with stratified analyses. Participants were grouped by age, BMI, comorbidities, and surgical history; within each stratum we calculated ORs and 95%CIs. Interaction terms tested effect modification, and forest plots illustrated heterogeneity among subgroups.

All computations were executed in R (http://www.R-project.org). Logistic models relied on glm, and forest plots were produced with ggplot2. Two-sided P-values below 0.05 were considered statistically significant.

## Results

### Baseline characteristics and independent association between blood lipids, inflammation and EM

**Figure 1:**
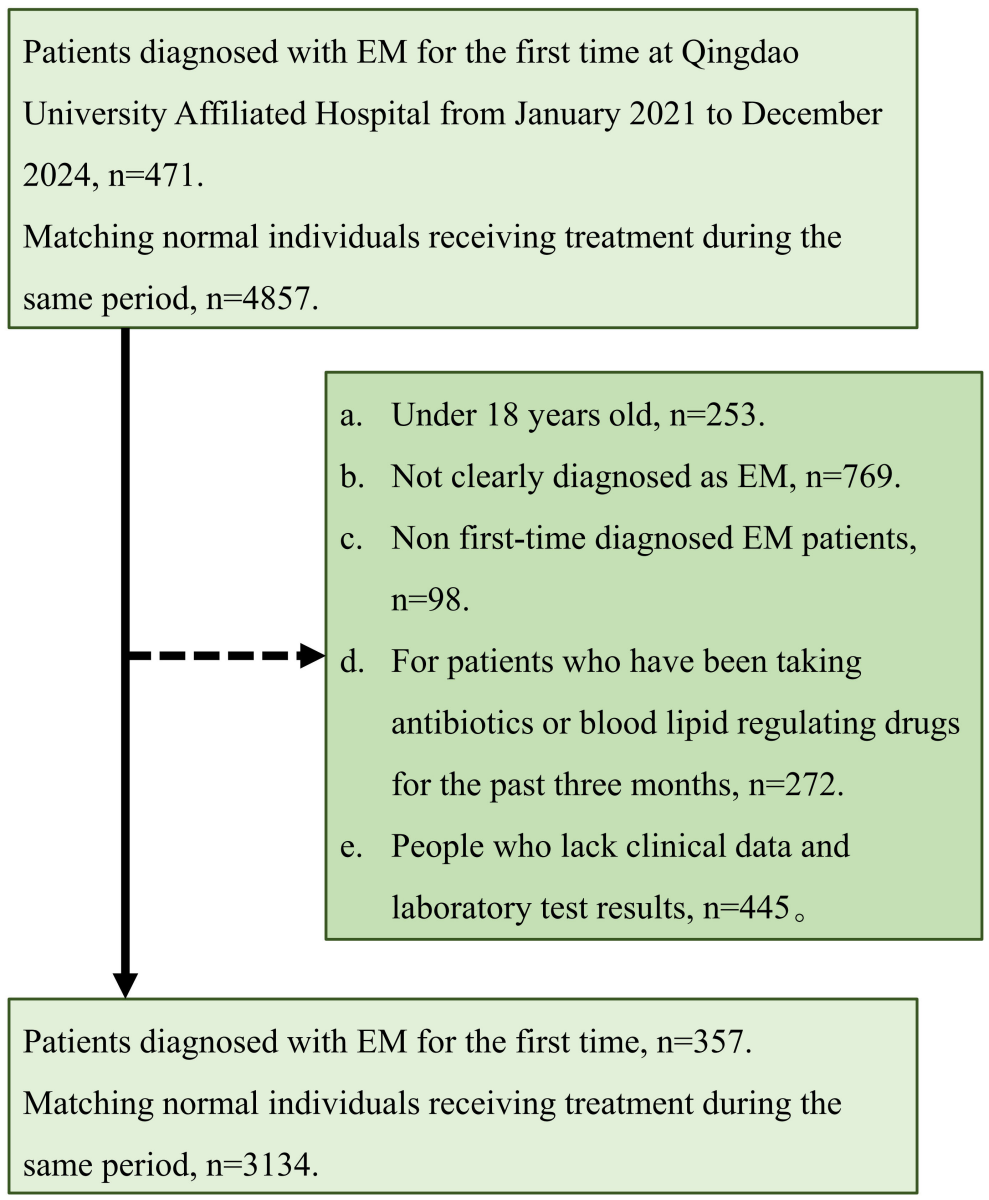
Research population inclusion and exclusion process.

This study enrolled 3,491 women with a mean age of 41.99 ± 9.33 years, of whom 357 (10.23%) were in the EM group and 3,134 (89.77%) in the non-EM group. The inclusion and exclusion flow is depicted in **Figure 1**. Significant differences were observed between the EM and non-EM groups in height, BMI, prevalence of overweight, menstrual regularity, menstrual flow, incidence of dysmenorrhea, HDL, TG, VLDL, NHHR, and inflammatory markers such as NLR and SII (all P < 0.05). Specifically, the EM group had lower HDL concentrations (1.48 ± 0.32 mmol/L, P < 0.001), higher TG concentrations (1.02 ± 0.68 mmol/L, P = 0.007), and elevated NHHR (2.39 ± 0.82, P < 0.001). Moreover, NLR (1.84 ± 0.56) and SII (451.34 ± 140.48) were higher than those in the non-EM group, indicating a potential increase in inflammatory status (**Table 1**).

Univariate logistic regression (**Supplementary Table 1**) showed that HDL was significantly inversely associated with EM (OR = 0.39), while TG (OR = 1.21), NHHR (OR = 1.57), and VLDL (OR = 1.81) were positively associated. Among inflammatory indicators, NLR (OR = 2.10), SII (OR = 1.01), and PLT also showed significant positive associations, with NLR having the strongest link. Multivariable logistic regression (**Table 2**) indicated that after adjusting for confounders, HDL remained a significant protective factor (OR = 0.45), whereas TG (OR = 1.21) and NHHR (OR = 1.54) were independent risk factors. NLR (OR = 2.04) and SII (OR = 1.01) also remained significantly associated with EM, suggesting that inflammation may be a mechanistic mediator between dyslipidemia and EM.

**Table 2:** EM multiple regression analysis.

Exposure	Model I OR (95%CI)Pvalue	Model II OR (95%CI) Pvalue	Model III OR (95%CI) Pvalue	Model IV OR (95%CI) Pvalue
HDL	0.39 (0.27, 0.56) <0.0001	0.38 (0.27, 0.55) <0.0001	0.46 (0.31, 0.69) 0.0002	0.45 (0.30, 0.67) 0.0001
LDL	1.06 (0.90, 1.24) 0.5010	1.05 (0.89, 1.23) 0.5601	1.12 (0.93, 1.34) 0.2344	1.10 (0.92, 1.32) 0.2987
VLDL	1.81 (1.08, 3.05) 0.0246	1.81 (1.07, 3.07) 0.0261	1.81 (1.00, 3.27) 0.0498	1.64 (0.89, 3.00) 0.1111
TC	0.94 (0.83, 1.07) 0.3641	0.94 (0.82, 1.06) 0.3043	1.00 (0.86, 1.15) 0.9787	0.98 (0.85, 1.14) 0.7965
TG	1.21 (1.05, 1.41) 0.0101	1.22 (1.05, 1.41) 0.0101	1.23 (1.04, 1.46) 0.0174	1.21 (1.02, 1.44) 0.0326
NHHR	1.57 (1.35, 1.82) <0.0001	1.58 (1.36, 1.84) <0.0001	1.54 (1.30, 1.83) <0.0001	1.54 (1.29, 1.84) <0.0001
LC	2.17 (1.78, 2.64) <0.0001	2.14 (1.76, 2.62) <0.0001	2.05 (1.62, 2.60) <0.0001	2.04 (1.60, 2.60) <0.0001
NC	2.60 (2.28, 2.97) <0.0001	2.59 (2.26, 2.96) <0.0001	2.55 (2.18, 2.99) <0.0001	2.51 (2.13, 2.95) <0.0001
PLT	1.01 (1.01, 1.02) <0.0001	1.01 (1.01, 1.02) <0.0001	1.01 (1.01, 1.02) <0.0001	1.01 (1.01, 1.02) <0.0001
PLR	1.00 (1.00, 1.01) 0.0390	1.00 (1.00, 1.01) 0.0243	1.00 (1.00, 1.01) 0.0509	1.00 (1.00, 1.01) 0.0387
NLR	2.10 (1.70, 2.60) <0.0001	2.21 (1.77, 2.75) <0.0001	2.01 (1.56, 2.59) <0.0001	2.04 (1.57, 2.65) <0.0001
SII	1.02 (1.01, 1.02) <0.0001	1.02 (1.01, 1.02) <0.0001	1.01 (1.01, 1.02) <0.0001	1.01 (1.01, 1.02) <0.0001

Model I no adjusted.

Model II adjusted for age(smooth), height(smooth), weight(smooth), BMI(smooth), overweight, history of cardiovascular and cerebrovascular diseases, history of diabetes and history of cancer.

Model III adjusted for history of open surgery, history of hysteroscopy and laparoscopic surgery, age at menarche(smooth), menstrual regularity, amount of menses and dysmenorrhea.

Model IV adjusted for age(smooth), height(smooth), weight(smooth), BMI(smooth), overweight, history of cardiovascular and cerebrovascular diseases, history of diabetes, history of cancer, history of open surgery, history of hysteroscopy and laparoscopic surgery, age at menarche(smooth), menstrual regularity, amount of menses and dysmenorrhea.

### Association between blood lipids and inflammatory markers

This study used multiple linear regression models to assess the associations between lipid markers (HDL, LDL, VLDL, TC, TG, NHHR) and inflammatory indicators (LC, NC, PLT, PLR, NLR, SII) (**Table 3**). Across both the unadjusted model (Model I) and the progressively adjusted models (Models II–IV), HDL showed a significant inverse correlation with most inflammatory indicators. In the fully adjusted model (Model IV), each 1 mmol/L increase in HDL was associated with a mean decrease of 0.38 in NC (95% CI, –0.45, –0.30; P < 0.0001), a decrease of 46.98 in SII (95% CI, –55.39, –38.57; P < 0.0001), a decrease of 0.15 in NLR (P < 0.0001), and negative associations with PLT and LC as well. Conversely, TG and NHHR were positively correlated with all inflammatory indicators: for each 1 mmol/L increase in TG, SII rose by 15.14 (95% CI, 10.50, 19.78; P < 0.0001); for each 1-unit increase in NHHR, SII rose by 28.45 (95% CI, 24.25, 32.64; P < 0.0001). LDL, VLDL, and TC also exhibited positive trends with several inflammatory markers, with VLDL showing the strongest associations—particularly with NC (β = 0.42, P < 0.0001) and SII (β = 36.77, P < 0.0001)—suggesting it may exert a greater influence in inflammation activation.

**Table 3:** Multiple regression analysis of inflammation.

Exposure	Model I β (95%CI) Pvalue	Model II β (95%CI) Pvalue	Model III β (95%CI) Pvalue	Model IV β (95%CI) Pvalue
LC				
HDL	-0.10 (-0.16, -0.05) <0.0001	-0.10 (-0.15, -0.05) <0.0001	-0.10 (-0.15, -0.05) 0.0001	-0.10 (-0.15, -0.05) 0.0002
LDL	0.06 (0.04, 0.09) <0.0001	0.06 (0.04, 0.09) <0.0001	0.07 (0.04, 0.09) <0.0001	0.07 (0.04, 0.09) <0.0001
VLDL	0.18 (0.10, 0.26) <0.0001	0.18 (0.10, 0.27) <0.0001	0.18 (0.09, 0.26) <0.0001	0.18 (0.09, 0.26) <0.0001
TC	0.03 (0.02, 0.05) 0.0005	0.03 (0.02, 0.05) 0.0004	0.04 (0.02, 0.06) 0.0003	0.04 (0.02, 0.05) 0.0003
TG	0.12 (0.10, 0.15) <0.0001	0.12 (0.10, 0.15) <0.0001	0.12 (0.10, 0.15) <0.0001	0.12 (0.10, 0.15) <0.0001
NHHR	0.11 (0.08, 0.13) <0.0001	0.10 (0.08, 0.13) <0.0001	0.10 (0.08, 0.13) <0.0001	0.10 (0.08, 0.13) <0.0001
NC				
HDL	-0.39 (-0.47, -0.31) <0.0001	-0.39 (-0.47, -0.31) <0.0001	-0.38 (-0.45, -0.30) <0.0001	-0.38 (-0.45, -0.30) <0.0001
LDL	0.13 (0.09, 0.16) <0.0001	0.13 (0.09, 0.16) <0.0001	0.13 (0.09, 0.17) <0.0001	0.13 (0.09, 0.17) <0.0001
VLDL	0.43 (0.30, 0.56) <0.0001	0.43 (0.30, 0.56) <0.0001	0.42 (0.29, 0.55) <0.0001	0.42 (0.29, 0.55) <0.0001
TC	0.04 (0.01, 0.07) 0.0039	0.04 (0.01, 0.07) 0.0040	0.05 (0.02, 0.08) 0.0015	0.05 (0.02, 0.08) 0.0018
TG	0.22 (0.18, 0.26) <0.0001	0.22 (0.18, 0.26) <0.0001	0.22 (0.18, 0.26) <0.0001	0.22 (0.17, 0.26) <0.0001
NHHR	0.26 (0.22, 0.30) <0.0001	0.26 (0.22, 0.30) <0.0001	0.26 (0.22, 0.30) <0.0001	0.26 (0.22, 0.29) <0.0001
PLT				
HDL	-11.27 (-16.29, -6.26) <0.0001	-11.42 (-16.43, -6.40) <0.0001	-10.50 (-15.50, -5.50) <0.0001	-10.67 (-15.67, -5.68) <0.0001
LDL	7.38 (4.97, 9.80) <0.0001	7.30 (4.88, 9.71) <0.0001	7.54 (5.13, 9.95) <0.0001	7.40 (5.00, 9.81) <0.0001
VLDL	13.36 (5.08, 21.64) 0.0016	13.25 (4.97, 21.53) 0.0017	12.86 (4.61, 21.11) 0.0023	12.46 (4.21, 20.72) 0.0031
TC	3.66 (1.75, 5.57) 0.0002	3.59 (1.68, 5.50) 0.0002	3.85 (1.95, 5.75) <0.0001	3.72 (1.82, 5.62) 0.0001
TG	7.56 (4.83, 10.30) <0.0001	7.49 (4.76, 10.22) <0.0001	7.46 (4.74, 10.18) <0.0001	7.34 (4.62, 10.07) <0.0001
NHHR	10.03 (7.52, 12.53) <0.0001	9.98 (7.48, 12.49) <0.0001	9.74 (7.24, 12.24) <0.0001	9.66 (7.16, 12.15) <0.0001
PLR				
HDL	-1.06 (-5.04, 2.91) 0.6002	-1.29 (-5.27, 2.68) 0.5237	-1.11 (-5.09, 2.88) 0.5864	-1.26 (-5.25, 2.73) 0.5354
LDL	-2.14 (-4.06, -0.22) 0.0287	-2.19 (-4.11, -0.27) 0.0253	-2.14 (-4.06, -0.22) 0.0289	-2.17 (-4.10, -0.25) 0.0267
VLDL	-7.51 (-14.06, -0.97) 0.0245	-7.85 (-14.40, -1.30) 0.0189	-7.30 (-13.86, -0.74) 0.0292	-7.72 (-14.29, -1.15) 0.0214
TC	-1.88 (-3.39, -0.37) 0.0146	-1.97 (-3.48, -0.46) 0.0108	-1.88 (-3.39, -0.36) 0.0150	-1.94 (-3.46, -0.43) 0.0119
TG	-5.32 (-7.48, -3.16) <0.0001	-5.33 (-7.49, -3.17) <0.0001	-5.32 (-7.49, -3.16) <0.0001	-5.35 (-7.52, -3.18) <0.0001
NHHR	-2.23 (-4.23, -0.23) 0.0288	-2.20 (-4.20, -0.21) 0.0306	-2.21 (-4.22, -0.21) 0.0301	-2.20 (-4.21, -0.20) 0.0311
NLR				
HDL	-0.15 (-0.20, -0.10) <0.0001	-0.15 (-0.20, -0.11) <0.0001	-0.15 (-0.19, -0.10) <0.0001	-0.15 (-0.19, -0.10) <0.0001
LDL	0.01 (-0.01, 0.03) 0.4593	0.01 (-0.01, 0.03) 0.4641	0.01 (-0.01, 0.03) 0.4120	0.01 (-0.01, 0.03) 0.4197
VLDL	0.07 (-0.00, 0.15) 0.0644	0.07 (-0.01, 0.15) 0.0688	0.07 (-0.00, 0.15) 0.0578	0.07 (-0.01, 0.15) 0.0721
TC	-0.01 (-0.03, 0.00) 0.1589	-0.01 (-0.03, 0.00) 0.1492	-0.01 (-0.03, 0.01) 0.1960	-0.01 (-0.03, 0.01) 0.1829
TG	0.02 (-0.01, 0.04) 0.1865	0.02 (-0.01, 0.04) 0.1856	0.02 (-0.01, 0.04) 0.2139	0.02 (-0.01, 0.04) 0.2276
NHHR	0.06 (0.04, 0.08) <0.0001	0.06 (0.04, 0.08) <0.0001	0.06 (0.04, 0.08) <0.0001	0.06 (0.03, 0.08) <0.0001
SII				
HDL	-48.78 (-57.31, -40.24) <0.0001	-49.22 (-57.76, -40.68) <0.0001	-46.66 (-55.08, -38.25) <0.0001	-46.98 (-55.39, -38.57) <0.0001
LDL	14.46 (10.30, 18.63) <0.0001	14.33 (10.16, 18.50) <0.0001	15.00 (10.90, 19.10) <0.0001	14.74 (10.64, 18.84) <0.0001
VLDL	38.84 (24.57, 53.10) <0.0001	38.50 (24.22, 52.78) <0.0001	38.14 (24.09, 52.19) <0.0001	36.77 (22.70, 50.83) <0.0001
TC	3.98 (0.68, 7.28) 0.0182	3.82 (0.52, 7.13) 0.0235	4.59 (1.34, 7.84) 0.0057	4.32 (1.07, 7.57) 0.0093
TG	15.86 (11.14, 20.57) <0.0001	15.75 (11.03, 20.47) <0.0001	15.43 (10.78, 20.07) <0.0001	15.14 (10.50, 19.78) <0.0001
NHHR	29.36 (25.10, 33.62) <0.0001	29.32 (25.06, 33.58) <0.0001	28.68 (24.48, 32.88) <0.0001	28.45 (24.25, 32.64) <0.0001

Model I no adjusted.

Model II adjusted for age(smooth), height(smooth), weight(smooth), BMI(smooth), overweight, history of cardiovascular and cerebrovascular diseases, history of diabetes and history of cancer.

Model III adjusted for history of open surgery, history of hysteroscopy and laparoscopic surgery, age at menarche(smooth), menstrual regularity, amount of menses and dysmenorrhea.

Model IV adjusted for age(smooth), height(smooth), weight(smooth), BMI(smooth), overweight, history of cardiovascular and cerebrovascular diseases, history of diabetes, history of cancer, history of open surgery, history of hysteroscopy and laparoscopic surgery, age at menarche(smooth), menstrual regularity, amount of menses and dysmenorrhea.

### Mediating role of inflammatory state in the association between blood lipids and EM

**Figure 2:**
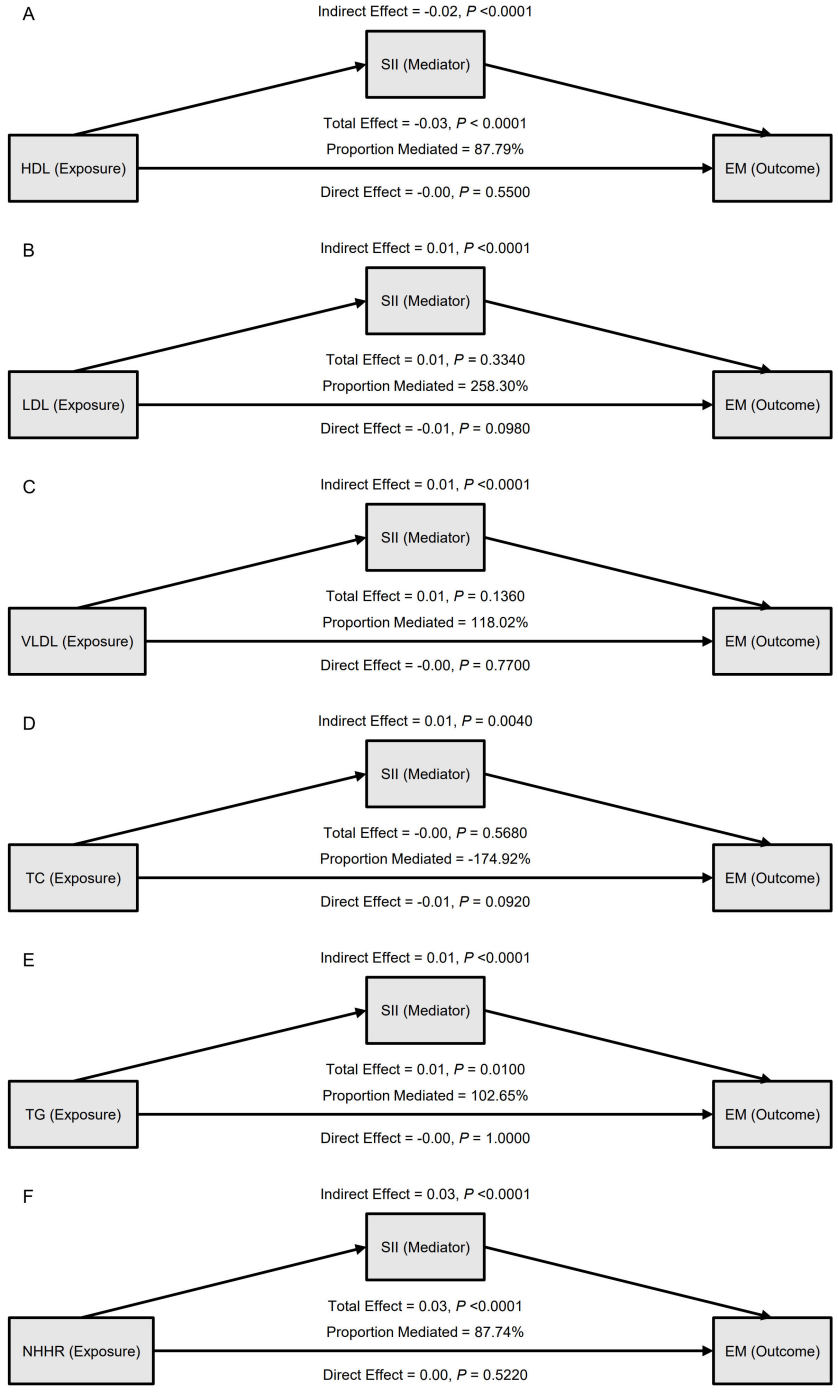
Mediation analysis of the association between blood lipid indicators and endometriosis (EM), with systemic inflammation index (SII) as the mediator. **(A)** HDL as the exposure; **(B)** LDL as the exposure; **(C)** VLDL as the exposure; **(D)** TC as the exposure; **(E)** TG as the exposure; **(F)** NHHR as the exposure. For each model, indirect, direct, and total effects are shown, along with the proportion mediated.

A mediation model adjusted for confounders was constructed with SII as the mediator to evaluate the role of inflammation in the link between dyslipidemia and EM (**Table 4**, **Figure 2**). The results showed that the effect of HDL on EM was primarily indirect through SII: the total effect was –0.03 (95% CI: -0.04, -0.01, P < 0.0001), the indirect effect was –0.02 (P < 0.0001), and the direct effect was not significant (P = 0.5500); the proportion mediated was 87.79%. TG and NHHR exhibited similar patterns: for TG, the total effect was 0.01 (P = 0.0100), the indirect effect 0.01 (P < 0.0001), and the proportion mediated 102.65%; for NHHR, the total effect was 0.03 (P < 0.0001), the indirect effect 0.03 (P < 0.0001), and the proportion mediated 87.74%. In contrast, although LDL, VLDL, and TC showed statistically significant indirect effects in some models, neither their total effects nor direct effects reached significance, suggesting a weaker role in the inflammation-mediated pathway. The path diagram (**Figure 2**) illustrates the proposed mechanism whereby SII mediates the lipid–EM relationship.

**Table 4:** Intermediary analysis.

Mediator: SII	Mediation effect, β (95%CI) Pvalue			
	Total effect	Indirect effect	Direct effect	Mediation
HDL	-0.03(-0.04, -0.01) <0.0001	-0.02(-0.03, -0.02) <0.0001	-0.00(-0.01, 0.01) 0.5500	87.79%
LDL	0.01(-0.01, 0.02) 0.3340	0.01(0.01, 0.02) <0.0001	-0.01(-0.02, 0.00) 0.0980	258.30%
VLDL	0.01(-0.00, 0.02) 0.1360	0.01(0.01, 0.01) <0.0001	-0.00(-0.01, 0.01) 0.7700	118.02%
TC	-0.00(-0.01, 0.01) 0.5680	0.01(0.00, 0.01) 0.0040	-0.01(-0.02, 0.00) 0.0920	-174.92%
TG	0.01(0.00, 0.02) 0.0100	0.01(0.01, 0.01) <0.0001	-0.00(-0.01, 0.01) 1.0000	102.65%
NHHR	0.03(0.02, 0.04) <0.0001	0.03(0.02, 0.03) <0.0001	0.00(-0.01, 0.01) 0.5220	87.74%

Adjust for: age(smooth), height(smooth), weight(smooth), BMI(smooth), overweight, history of cardiovascular and cerebrovascular diseases, history of diabetes, history of cancer, history of open surgery, history of hysteroscopy and laparoscopic surgery, age at menarche(smooth), menstrual regularity, amount of menses and dysmenorrhea.

In the external validation set, the mediation findings were broadly consistent (**Supplementary Tables 2, 3**, **Supplementary Figure 1**). For HDL, the total effect was –0.13 (95% CI: –0.17, –0.10, P < 0.0001), with an indirect effect of –0.06 and a direct effect of –0.07 (both P < 0.0001), yielding a proportion mediated of 45.09%; for NHHR, the proportion mediated was 25.77%. In this cohort, TG showed an indirect effect of –0.03 (P = 0.0020) opposite in direction to its total effect, implying potential influence from population characteristics or other confounders. The mechanism whereby lower HDL and higher NHHR increase EM risk through the high-inflammation state reflected by SII was validated in both the main analysis and the external validation set, supporting a key mediating role for systemic inflammation in the pathway from dyslipidemia to EM.

### Smooth fitting curve and prediction model construction and validation

**Figure 3:**
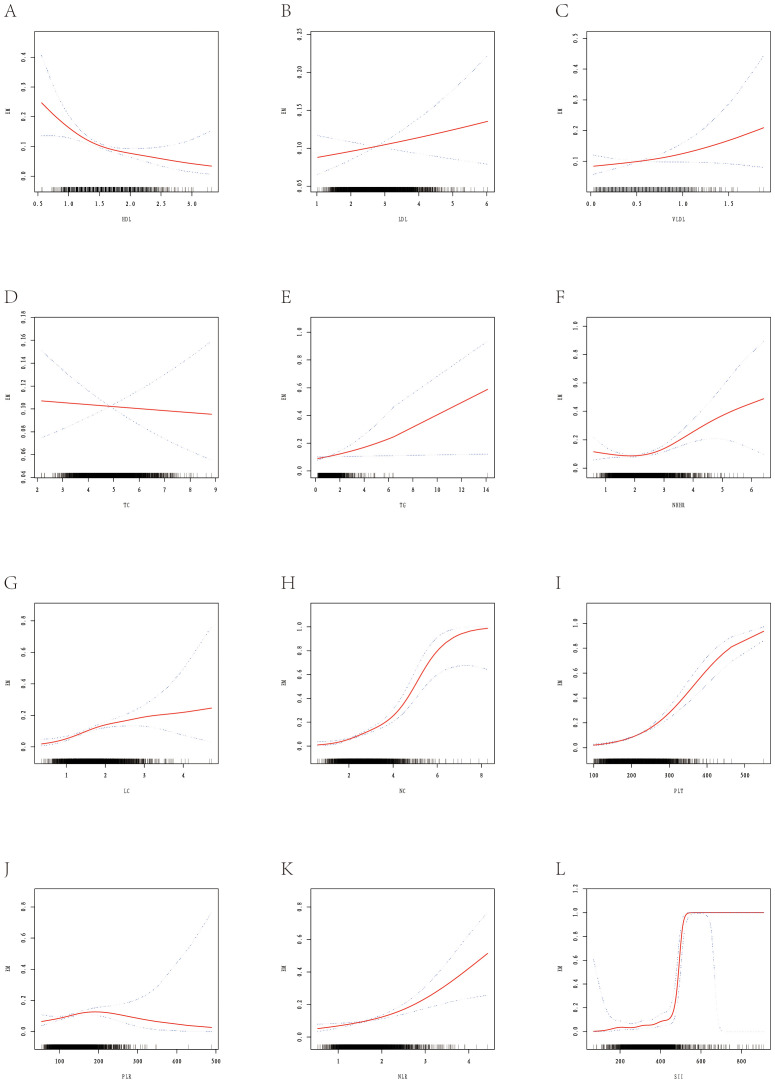
Smooth fitting curves illustrating the association between selected metabolic-inflammatory indicators and the risk of EM. The red solid line represents the smoothed log-odds estimated with a generalized additive model; blue dashed lines denote the 95% confidence intervals. Rug plots on the x-axis show individual data distributions. **(A)** Shows the smooth-fitting curve between HDL and EM. **(B)** Shows the smooth-fitting curve between LDL and EM. **(C)** Shows the smooth-fitting curve between VLDL and EM. **(D)** Shows the smooth-fitting curve between TC and EM. **(E)** Shows the smooth-fitting curve between TG and EM. **(F)** Shows the smooth-fitting curve between NHHR and EM. **(G)** Shows the smooth-fitting curve between LC and EM. **(H)** Shows the smooth-fitting curve between NC and EM. **(I)** Shows the smooth-fitting curve between PLT and EM. **(J)** Shows the smooth-fitting curve between PLR and EM. **(K)** Shows the smooth-fitting curve between NLR and EM. **(L)** Shows the smooth-fitting curve between SII and EM.

**Figure 4:**
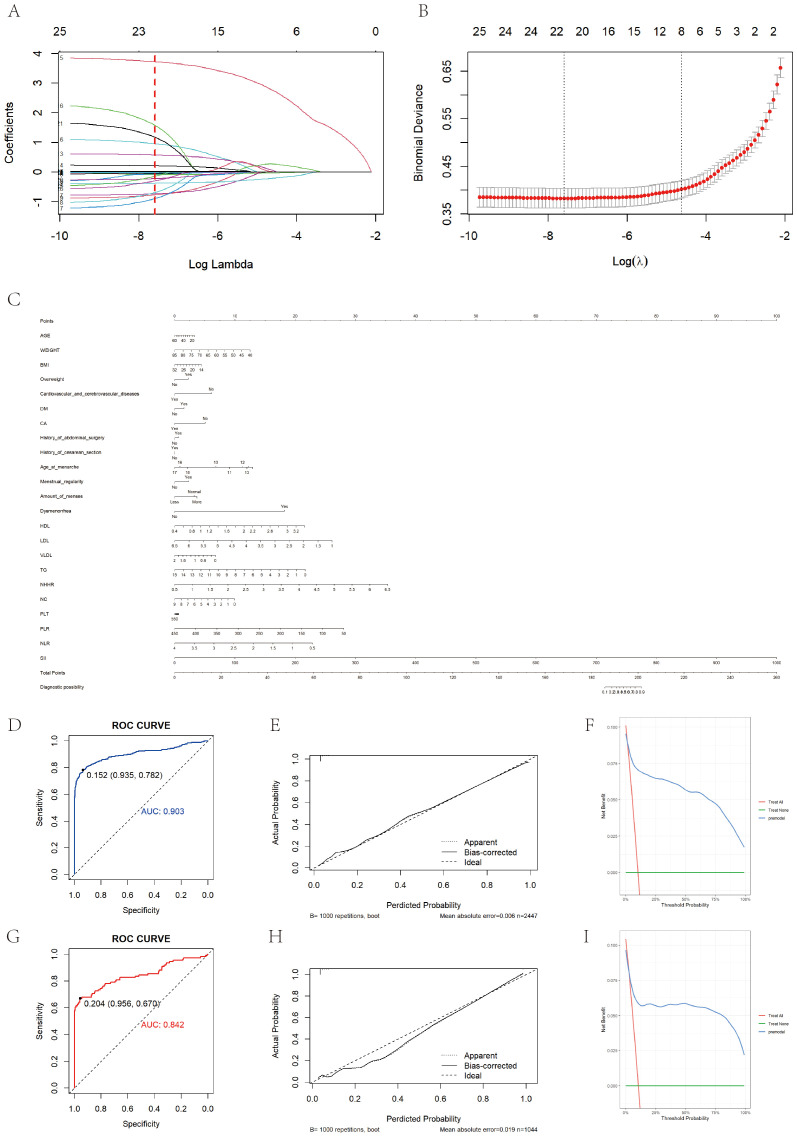
Prediction model construction and evaluation. **(A)** Shows the variation characteristics of the coefficient of variation. Each curve in the graph represents the coefficient variation of each variable. The vertical axis represents coefficient values, the lower horizontal axis represents log (λ), and the upper horizontal axis represents the number of non-zero coefficients in the model at this time. **(B)** Shows the process of selecting the optimal value of parameter λ in the LASSO regression model using the ten fold cross validation method. **(C)** Is a bar chart showing the diagnosis of EM based on clinical symptoms and laboratory test results. When using a column chart, use a ruler to draw a vertical line between the target variable and the dot scale at the top of the chart to determine the contribution of each variable to the total score. Add up the number of points for each variable, and then draw a vertical line from the total score at the bottom of the bar chart to the disease outcome to determine the estimated result. **(D)** Shows the ROC curve of the training set based on a column chart, with an AUC value of 0.903. **(G)** Shows the ROC curve of the validation set based on a column chart, with an AUC value of 0.842. **(E, H)** Show the calibration curves of the training set and internal validation set based on column charts, respectively. The dashed line represents the ideal reference line, where the predicted probability matches the observed survival rate, while the solid line is used to calculate the performance of the bar chart. The closer the solid line is to the dashed line, the more accurate the model prediction will be. **(F)** Shows the DCA curve of the training set based on a column chart. **(I)** Shows the DCA curve drawn based on a column chart for internal validation.

**Figure 5:**
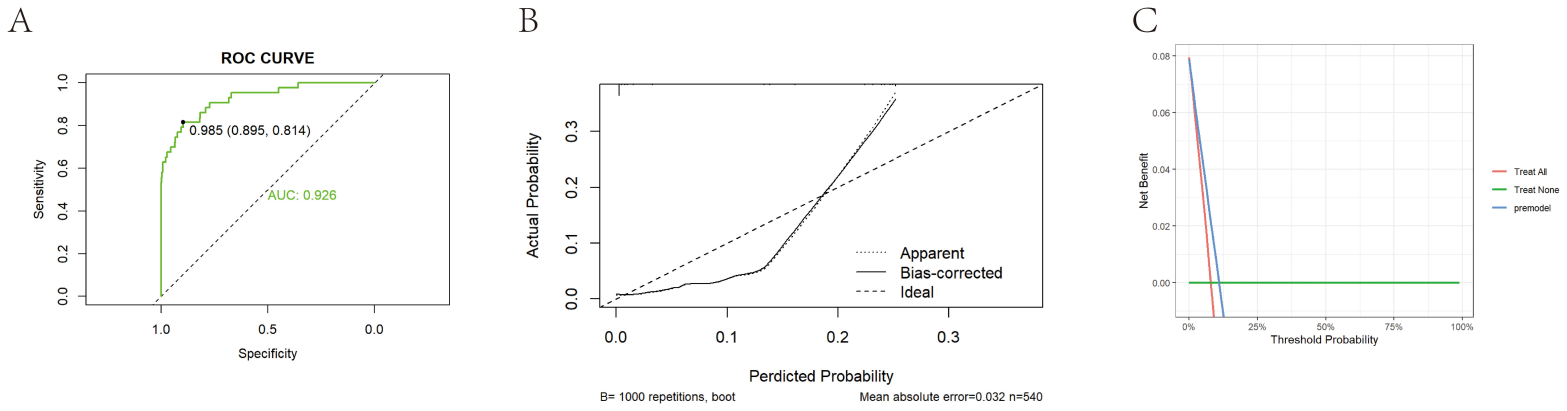
External validation of prediction model construction. **(A)** Shows the ROC curve of the external validation set based on a bar chart, with an AUC value of 0.926. **(B)** Shows the calibration curve of the external validation set based on a bar chart. The dashed line represents the ideal reference line, where the predicted probability matches the observed survival rate, while the solid line is used to calculate the performance of the bar chart. The closer the solid line is to the dashed line, the more accurate the model prediction will be. **(C)** Shows the DCA curve of an external training set based on a bar chart.

This study assessed the nonlinear associations between lipid indices and EM risk (**Supplementary Table 4**, **Figure 3**) and developed an early risk-prediction model (**Supplementary Tables 5–7**, **Figures 4**, **5**). Nonlinear analysis revealed a strong inverse relationship between HDL and EM risk, with risk decreasing steadily as HDL increased; a threshold of 1.34 mmol/L was identified, below which risk rose sharply (**Supplementary Table 4**, **Figure 3A**). Both TG and NHHR were positively correlated with risk, and NHHR showed a rapid increase in risk beyond an inflection at 2.08 (**Figures 3E, F**). LDL, VLDL, and TC exhibited no notable inflection points, while inflammatory markers (NLR, SII, etc.) displayed monotonic upward trends (**Figures 3G–L**).

The study population was then randomly divided into training and validation sets, which were balanced in demographic characteristics, lipid measures, and inflammatory markers (**Supplementary Table 5**). A LASSO–logistic regression with 10-fold cross-validation in the training set identified 26 important variables (**Figures 4A, B**), which were used to construct a multivariable logistic model and a nomogram (**Figure 4C**). Key predictors included HDL, NHHR, dysmenorrhea, PLR, and SII (**Supplementary Tables 6, 7**). The model achieved AUCs of 0.903 in the training set, 0.842 in the validation set, and 0.926 in an independent external cohort (**Figures 4D**, 4G, 5A); calibration curves demonstrated close agreement between predicted probabilities and observed risks (**Figures 4E, H**, **5B**). Decision-curve analysis showed meaningful clinical net benefits across a probability-threshold range of 0.05–0.25 (**Figures 4F, I**, **5C**).

### Stratified and subgroup analyses for robustness verification

Extensive subgroup stratification and interaction testing (**Supplementary Tables 8–14**, **Supplementary Figures 2–13**) showed that HDL was a significant protective factor against endometriosis (EM) in every subgroup (OR = 0.27–0.57), with an even stronger effect among overweight women, those without prior surgery, and those with heavy menstrual flow, while most interaction terms were non-significant, underscoring HDL’s broad applicability; LDL was not associated with EM overall (OR = 1.06, P = 0.501) but showed a positive link in overweight women (OR = 1.24, P = 0.044) with a significant LDL-overweight interaction (P = 0.010); VLDL remained positively associated across subgroups, especially in overweight women, those with open abdominal surgery, and those with heavy flow; total cholesterol was generally null, except for a mildly protective trend in non-overweight individuals (OR = 0.82, P = 0.048); triglycerides were a clear risk factor overall, with stronger effects in overweight women and those with prior abdominal or laparoscopic surgery (OR = 1.37–1.58); NHHR was the most consistent risk marker, remaining positively associated in all subgroups and reaching its highest odds ratios (= 1.9–2.3) in overweight and heavy-flow women, with a significant NHHR-overweight interaction (P = 0.011), altogether indicating that low HDL and elevated TG and NHHR are more tightly linked to EM risk in metabolically stressed or surgically exposed women and can thus inform individualized, metabolism-oriented risk assessment and prevention strategies.

## Discussion

Using large-scale case–control data, this study systematically evaluated the relationships between multiple lipid markers and EM risk, examined the mediating role of systemic inflammation, and developed a prediction model to facilitate early risk identification. The findings indicate that lower HDL, together with higher TG and NHHR, are independent risk factors for EM, with these associations being especially pronounced in women who are overweight or have a history of abdominal surgery.

Previous epidemiological studies examining the relationship between blood-lipid concentrations and EM have generally involved small samples, and their conclusions remain inconsistent. Nahar et al. (18) were the first to report in South Asian women that EM patients typically display a characteristic dyslipidemic pattern—reduced HDL combined with elevated TC, LDL, and TG—yet the underlying pathological mechanisms were not further explored. Two subsequent cross-sectional studies using data from the National Health and Nutrition Examination Survey (NHANES) further expanded the evidence base. Chen et al. (19) reported that each 1 mmol/L increase in remnant cholesterol doubled the risk of EM, while Lin et al. (20) demonstrated a linear positive association between the triglyceride–glucose index (TyG) and its derivative indices and EM. However, none of these studies incorporated systemic inflammation into their analytic framework. Our work is the first, within a large case–control design, to place lipid parameters, systemic inflammation, and EM in a single analytical model and to quantify precisely, via the SII, the proportion of inflammation mediating the link—thus completing the causal chain of “metabolic disturbance leading to inflammatory activation, which subsequently drives EM progression”. We showed that HDL concentrations are markedly lower in EM patients than in controls and that HDL exhibits a steady inverse relationship with EM risk, suggesting a protective role (21, 22). In contrast, plasma TG and NHHR are significantly elevated and positively associated with EM, indicating that lipid-metabolic imbalance can create a chronic inflammatory milieu (7–9, 23, 24) that supports the survival and invasion of ectopic lesions. Notably, NHHR captures overall exposure to atherogenic lipoproteins more comprehensively than single lipid measures. Our findings further validate its value in EM risk prediction and align with the view of EM as a “chronic inflammatory disease”.

The SII serves as the crucial link between blood-lipid markers and endometriosis risk. HDL’s protective effect—and the risk increments associated with TG and NHHR—act predominantly through SII, a mediation pattern that was replicated in the external validation cohort. Notably, the mediation proportion for TG reached 103%, constituting an inconsistent mediation, indicating that the indirect effect of TG via SII opposes its direct effect. In the external validation cohort, this inverse indirect effect for TG may partly reflect differences in baseline population characteristics between the two cohorts. For example, the mean age in both centers exceeded 40 years, which is higher than in most epidemiological studies of EM and likely reflects the hospital-based surgical inpatient recruitment pattern. Such an age distribution could alter lipid metabolism and systemic inflammatory responses, especially around the perimenopausal transition, thereby influencing the mediation pathway. Although some between-group differences in baseline characteristics reached statistical significance, their clinical relevance may be limited. Nonetheless, residual confounding from gynecologic comorbidities, lifestyle, or unmeasured metabolic factors cannot be excluded. These findings imply that elevated TG often accompanies lipid fractions that seem partially protective (25, 26), but once inflammation is accounted for, this apparent benefit disappears, exposing TG’s inherent risk. Spline curves further showed that EM risk rises steeply at HDL < 1.34 mmol/L, NHHR > 2.08, and TG > 1.7 mmol/L—thresholds close to those in international cardiovascular and metabolic-disease guidelines (22, 23, 27–29), making them operational across disease contexts. This relationship has not been systematically reported before, filling a gap in metabolic-factor research on EM pathogenesis. In EM’s high-estrogen milieu, over-activated estrogen receptor alpha/beta (ER-α/β) disrupt hepatic genes such as ATP-binding cassette transporter A1 (BCA1) and lipoprotein lipase (LPL), flipping the normally protective “HDL-high, TG-low” profile into HDL dysfunction and TG accumulation; simultaneously, the estrogen receptor-alpha - signal transducer and activator of transcription 3 (ER-α–STAT3) axis drives macrophage polarization from resolutive M2 to pro-inflammatory M1 phenotypes (30, 31). Single-cell sequencing reveals that, within the HDL-depleted milieu of endometriotic lesions, metabolically impaired M2-like macrophages lose scavenger receptor class B type 1/ATP-binding cassette transporter G1 (SR-B1/ABCG1)-mediated cholesterol efflux, leading to cholesterol-crystal activation of the NLR family pyrin domain-containing 3 (NLRP3) inflammasome, while concomitant elevations in triglycerides and remnant cholesterol amplify interleukin-1 beta (IL-1β) release and thereby fuel angiogenesis and nerve infiltration (32–34). Meanwhile, lipid peroxidation products (malondialdehyde, 4-hydroxynonenal) in EM peritoneal fluid are markedly elevated, both intensifying the oxidative-stress–inflammation loop and potentially suppressing NLRP3, offering a biological explanation for the TG-SII mediation proportion > 100% (35, 36). Estrogen-induced lipid dysregulation, macrophage metabolic reprogramming, and oxidative stress interlock to form a self-perpetuating pathological loop. Overactive estrogen receptors diminish HDL function and elevate TG and NHHR, which then activate the NLRP3 inflammasome and drive excessive IL-1β release, sustaining lesion growth and underscoring the pivotal role of the lipid–inflammation axis in endometriosis.

Although our results support a mediating role of systemic inflammation, we were unable to directly evaluate whether higher inflammatory indices correlated with more severe clinical symptoms (e.g., dysmenorrhea, infertility) or greater anatomical lesion extent, as standardized symptom scoring and rAFS staging data were not consistently available for all participants. Future prospective studies incorporating these parameters are needed to clarify whether inflammation intensity parallels EM severity. In addition, inflammation could also be a downstream consequence of established EM lesions. Local immune activation within ectopic endometrial tissue may release cytokines and chemokines into the systemic circulation, thereby elevating inflammatory indices independently of pre-existing metabolic disturbances. This reverse-causality pathway should be considered as an alternative explanation and warrants confirmation in longitudinal or interventional studies. To place our findings in a broader context, we have drawn on mechanistic concepts from atherosclerosis research, where lipid-inflammation interactions are well established. Analogous processes such as HDL functional impairment, triglyceride-rich lipoprotein accumulation and cholesterol crystal-induced inflammasome activation may also occur in EM, albeit within a distinct hormonal and tissue microenvironment. Recognizing both the parallels and differences between these two chronic inflammatory conditions may provide novel avenues for translational research.

The immune-inflammatory ratios and the lipid-inflammation-based prediction model identified in this study may have potential applications for both clinicians and researchers. Clinically, these indices could serve as inexpensive and easily obtainable tools for early identification of women at elevated risk of endometriosis, for preoperative risk stratification, and for monitoring postoperative recurrence or treatment response. The nomogram could be incorporated into clinical decision-support systems after further validation. From a research perspective, these findings offer a framework for exploring the lipid-immune axis as a mechanistic bridge linking metabolism and inflammation in endometriosis, and for developing combined lipid-lowering and anti-inflammatory interventions. Future longitudinal and multi-omics studies are warranted to verify the predictive performance of these indices and elucidate their molecular basis.

For risk prediction, this study used LASSO regression to screen key variables and, together with a nomogram, built an EM risk-prediction model that combines strong discriminative power with intuitive visualization. The model demonstrated high accuracy and stability in the training set, validation set, and an independent external cohort, thereby filling the practical gap created by the lack of usable early risk-assessment tools in the EM field. The model notably incorporates NHHR, an emerging index that captures both metabolic load and inflammatory status (37), which markedly enhanced its discriminative capacity and broadened the scope of traditional risk assessment. Moreover, subgroup and interaction analyses identified BMI as a significant effect modifier: in overweight individuals, the associations of HDL, TG, and NHHR with EM were more pronounced, suggesting that a patient’s metabolic background may amplify the impact of dyslipidemia on inflammation and EM risk. Certain lipid markers (e.g., TG) also showed stronger risk effects in women with specific clinical traits, such as a surgical history or heavy menstrual flow, indicating that lipid-based risk evaluation should integrate metabolic status and gynecological characteristics to enable more precise stratified management and intervention strategies.

Despite the advantages of a large sample size, a systematic analytical framework, and external validation, this study has several limitations. First, detailed information on the anatomical subtype of endometriosis was incomplete in our retrospective records. Surgical and pathological reports were not uniformly available, precluding reliable classification into ovarian, peritoneal, or deep infiltrating types. As a result, we did not perform subtype-specific analyses. Future prospective studies with comprehensive intraoperative documentation are warranted to determine whether the observed lipid–inflammation associations are consistent across endometriosis subtypes. Second, although certain baseline characteristics in **Table 1** showed statistical significance, the absolute mean differences for some variables were <0.1 and may be within the assay’s standard error, suggesting limited clinical relevance. We have now provided SE values for key laboratory indices to allow readers to assess whether observed differences exceed expected measurement variability. Third, most inflammatory indicators were derived from routine hematological parameters and simple composite indices, which may not fully reflect the inflammatory status within the tissue microenvironment. Finally, mechanistic evidence is still lacking; further research is required to clarify the molecular pathways that connect dyslipidemia, inflammation, and EM.

## Conclusion

This study offers a new perspective on how metabolic dysregulation promotes EM and underscores the pivotal role of systemic inflammation. Using large-scale, multi-cohort case-control data, we applied causal mediation analysis to systematically verify that the SII mediates the pathways linking key lipid markers, including HDL-C, TG, and NHHR, to EM risk. The findings support a mechanistic chain whereby dyslipidemia triggers inflammatory activation, which in turn elevates EM risk.

Beyond methodological innovation, our results have direct clinical implications. The identified thresholds (HDL-C<1.34 mmol/L, TG>1.7 mmol/L, NHHR>2.08) are close to those used in cardiovascular and metabolic disease guidelines and can be readily obtained from routine laboratory tests. The SII, also derivable from standard blood counts, enables integration of metabolic and inflammatory status into individualized EM risk assessment. In practice, these parameters may help clinicians identify women, particularly those who are overweight or have specific gynecological histories, who are at elevated risk and could benefit from earlier metabolic modulation and anti-inflammatory interventions. This approach bridges statistical modelling and clinical decision-making, providing a feasible pathway toward targeted prevention and improved patient outcomes in EM.

## Data Availability

The original contributions presented in the study are included in the article/**Supplementary Material**. Further inquiries can be directed to the corresponding author.

## Author’s note

This study is the first to suggest the potential causal chain “dyslipidemia→SII-mediated inflammation→endometriosis. For endometriosis, we identified potential risk thresholds—HDL < 1.34 mmol/L, TG > 1.7 mmol/L, NHHR > 2.08—and assessed NHHR as a possible predictive marker.We developed a LASSO-based nomogram (AUC 0.84–0.93) that may support personalized lipid-modulating and antiinflammatory strategies to prevent or manage endometriosis.
