# Supplementary material for: Impact of the lipid–inflammation axis on endometriosis risk: a multicenter case–control study using mediation analysis
Source: Front Endocrinol (Lausanne). 2025 Nov 13;16:1661264. doi: 10.3389/fendo.2025.1661264 (PMC12628710; doi:10.3389/fendo.2025.1661264)
Supplement: Supplementary file 1 [file DataSheet1.pdf]

Table

Supplementary table 1. EM single factor analysis

	Mean±SD / N(%)	EM<br>OR (95%CI) P value
Age, years	41.99 ± 9.33	0.99 (0.98, 1.00) 0.0739
Height, cm	161.25 ± 4.72	0.95 (0.93, 0.97) <0.0001
Weight, kg	62.40 ± 7.29	1.00 (0.99, 1.02) 0.8943
BMI, kg/m^2	24.02 ± 2.81	1.05 (1.01, 1.10) 0.0088
Overweight, n (%)		
No	1734 (49.67%)	Reference
Yes	1757 (50.33%)	1.36 (1.09, 1.69) 0.0067
History of cardiovascular and cerebrovascular diseases, n (%)		
No	3309 (94.79%)	Reference
Yes	182 (5.21%)	0.50 (0.26, 0.95) 0.0339
History of diabetes, n (%)		
No	3325 (95.24%)	Reference
Yes	166 (4.76%)	0.67 (0.37, 1.22) 0.1943
History of cancer, n (%)		
No	3445 (98.68%)	Reference
Yes	46 (1.32%)	0.40 (0.10, 1.64) 0.2010
History of open surgery, n (%)		
No	2240 (64.16%)	Reference
Yes	1251 (35.84%)	1.01 (0.81, 1.27) 0.9009
History of hysteroscopy and laparoscopic surgery, n (%)		
No	2526 (72.36%)	Reference
Yes	965 (27.64%)	0.96 (0.75, 1.23) 0.7375
Age at menarche, years	13.92 ± 1.35	1.02 (0.94, 1.10) 0.6638
Menstrual regularity, n (%)		
Erratical	808 (23.15%)	Reference
Rule	2683 (76.85%)	1.78 (1.32, 2.40) 0.0002
Amount of menses, n (%)		
Less	131 (3.75%)	Reference
Normal	2983 (85.45%)	1.07 (0.58, 1.96) 0.8317
More	377 (10.80%)	1.69 (0.88, 3.27) 0.1169
Dysmenorrhea, n (%)		
No	2986 (85.53%)	Reference
Yes	505 (14.47%)	11.99 (9.43, 15.25) <0.0001
HLD, mmol/L	1.56 ± 0.33	0.39 (0.27, 0.56) <0.0001
LDL, mmol/L	2.74 ± 0.69	1.06 (0.90, 1.24) 0.5010
VLDL, mmol/L	0.55 ± 0.20	1.81 (1.08, 3.05) 0.0246

TC, mmol/L	4.85 ± 0.88	0.94 (0.83, 1.07) 0.3641
TG, mmol/L	0.93 ± 0.61	1.21 (1.05, 1.41) 0.0101
NHHR	2.19 ± 0.66	1.57 (1.35, 1.82) <0.0001
LC, 10^9/L	1.67 ± 0.51	2.17 (1.78, 2.64) <0.0001
NC, 10^9/L	2.68 ± 0.80	2.60 (2.28, 2.97) <0.0001
PLT, 10^9/L	215.49 ± 50.58	1.01 (1.01, 1.02) <0.0001
PLR	136.96 ± 39.95	1.00 (1.00, 1.01) 0.0390
NLR	1.67 ± 0.46	2.10 (1.70, 2.60) <0.0001
SII	348.84 ± 87.35	1.02 (1.01, 1.02) <0.0001

Supplementary table 2. Baseline features of external validation set

	Total(n=540) Mean±SD / N(%)	No-EM(n=497) Mean±SD / N(%)	EM(n=43) Mean±SD / N(%)	P value
Age, years	46.16 ± 7.22	46.44 ± 7.22	42.88 ± 6.42	0.002
Height, cm	160.87 ± 4.89	160.86 ± 4.88	160.98 ± 5.14	0.879
Weight, kg	62.32 ± 8.78	62.01 ± 8.66	65.98 ± 9.51	0.004
BMI, kg/m^2	24.08 ± 3.18	23.96 ± 3.14	25.44 ± 3.26	0.003
Overweight, n (%)				0.018
No	294 (54.44%)	278 (55.94%)	16 (37.21%)	
Yes	246 (45.56%)	219 (44.06%)	27 (62.79%)	
History of cardiovascular and cerebrovascular diseases, n (%)				0.913
No	513 (95.00%)	472 (94.97%)	41 (95.35%)	
Yes	27 (5.00%)	25 (5.03%)	2 (4.65%)	
History of diabetes, n (%)				0.971
No	527 (97.59%)	485 (97.59%)	42 (97.67%)	
Yes	13 (2.41%)	12 (2.41%)	1 (2.33%)	
History of cancer, n (%)				0.810
No	530 (98.15%)	488 (98.19%)	42 (97.67%)	
Yes	10 (1.85%)	9 (1.81%)	1 (2.33%)	
History of open surgery, n (%)				0.062
No	370 (68.52%)	346 (69.62%)	24 (55.81%)	
Yes	170 (31.48%)	151 (30.38%)	19 (44.19%)	
History of hysteroscopy and laparoscopic surgery, n (%)				0.012
No	469 (86.85%)	437 (87.93%)	32 (74.42%)	
Yes	71 (13.15%)	60 (12.07%)	11 (25.58%)	
Age at menarche, years	13.92 ± 1.45	13.96 ± 1.46	13.51 ± 1.30	0.052
Menstrual regularity, n (%)				0.139

Erratical	167 (30.93%)	158 (31.79%)	9 (20.93%)	
Rule	373 (69.07%)	339 (68.21%)	34 (79.07%)	
Amount of menses, n (%)				0.620
Less	20 (3.70%)	19 (3.82%)	1 (2.33%)	
Normal	44 (8.15%)	39 (7.85%)	5 (11.63%)	
More	476 (88.15%)	439 (88.33%)	37 (86.05%)	
Dysmenorrhea, n (%)				0.051
No	158 (29.26%)	151 (30.38%)	7 (16.28%)	
Yes	382 (70.74%)	346 (69.62%)	36 (83.72%)	
HLD, mmol/L	1.60 ± 0.29	1.64 ± 0.26	1.11 ± 0.23	<0.001
LDL, mmol/L	2.67 ± 0.59	2.66 ± 0.60	2.82 ± 0.54	0.097
VLDL, mmol/L	0.36 ± 0.22	0.35 ± 0.20	0.53 ± 0.32	<0.001
TC, mmol/L	4.63 ± 0.75	4.65 ± 0.75	4.45 ± 0.80	0.104
TG, mmol/L	0.99 ± 0.58	0.94 ± 0.55	1.55 ± 0.69	<0.001
NHHR	1.96 ± 0.54	1.87 ± 0.43	3.06 ± 0.49	<0.001
LC, 10^9/L	1.92 ± 0.50	1.93 ± 0.49	1.83 ± 0.57	0.230
NC, 10^9/L	3.08 ± 1.12	2.96 ± 0.94	4.46 ± 1.88	<0.001
PLT, 10^9/L	262.02 ± 61.28	256.83 ± 56.71	322.00 ± 78.82	<0.001
PLR	142.67 ± 41.61	138.92 ± 38.24	186.08 ± 53.48	<0.001
NLR	1.67 ± 0.79	1.58 ± 0.54	2.71 ± 1.84	<0.001
SII	429.95 ± 219.63	393.96 ± 109.43	845.90 ± 534.02	<0.001

Supplementary table 3. Mediation analysis of external validation set

Mediator: SII	Mediation effect, β (95%CI) Pvalue			
	Total effect	Indirect effect	Direct effect	Mediation
HDL	-0.13(-0.17, -0.10) <0.0001	-0.06(-0.08, -0.05) <0.0001	-0.07(-0.11, -0.03) <0.0001	45.09%
LDL	0.03(0.00, 0.07) 0.0420	0.01(-0.01, 0.03) 0.4220	0.03(-0.00, 0.05) 0.0580	22.25%
VLDL	0.07(0.04, 0.10) <0.0001	0.01(0.00, 0.02) 0.0340	0.06(0.03, 0.09) <0.0001	15.82%
TC	0.06(0.03, 0.11) <0.0001	0.02(0.01, 0.04) <0.0001	0.04(0.01, 0.08) <0.0001	37.48%
TG	-0.01(-0.05, 0.02) 0.4320	-0.03(-0.05, -0.01) 0.0020	0.02(-0.01, 0.05) 0.3080	202.98%
NHHR	0.22(0.17, 0.26) <0.0001	0.06(0.04, 0.08) <0.0001	0.16(0.12, 0.20) <0.0001	25.77%

Adjust for: age(smooth), height(smooth), weight(smooth), BMI(smooth), overweight, history of cardiovascular and cerebrovascular diseases, history of diabetes, history of cancer, history of open surgery, history of hysteroscopy and laparoscopic surgery, age at menarche(smooth), menstrual

regularity, amount of menses and dysmenorrhea.

Supplementary table 4. Analysis of saturation threshold effect

Exposure	HDL OR (95%CI) Pvalue	LDL OR (95%CI) Pvalue	VLDL OR (95%CI) Pvalue	TC OR (95%CI) Pvalue	TG OR (95%CI) Pvalue	NHHR OR (95%CI) Pvalue
Model I						
One linear effect	0.45 (0.30, 0.67) 0.0001	1.10 (0.92, 1.32) 0.2987	1.64 (0.89, 3.00) 0.1111	0.98 (0.85, 1.14) 0.7965	1.21 (1.02, 1.44) 0.0326	1.54 (1.29, 1.84) <0.0001
Model II						
Break point (k)	1.34	2.04	0.79	4.15	1.99	2.08
<k segment effect 1	0.14 (0.04, 0.53) 0.0034	1.40 (0.46, 4.22) 0.5558	1.05 (0.46, 2.42) 0.8994	1.59 (0.82, 3.09) 0.1699	1.32 (0.98, 1.78) 0.0667	0.69 (0.42, 1.12) 0.1314
>k segment effect 2	0.63 (0.36, 1.08) 0.0936	1.08 (0.87, 1.33) 0.5086	4.77 (1.08, 21.08) 0.0391	0.90 (0.74, 1.08) 0.2628	1.10 (0.77, 1.58) 0.6107	2.06 (1.62, 2.61) <0.0001
Effect difference between 2 and 1	4.33 (0.86, 21.68) 0.0749	0.77 (0.23, 2.57) 0.6713	4.53 (0.64, 31.86) 0.1294	0.56 (0.26, 1.20) 0.1391	0.83 (0.49, 1.42) 0.4987	3.00 (1.59, 5.65) 0.0007
Predicted value of equation at break point	-2.14 (-2.33, -1.96)	-2.21 (-2.40, -2.01)	-2.17 (-2.39, -1.95)	-2.02 (-2.20, -1.83)	-1.85 (-2.13, -1.56)	-2.52 (-2.70, -2.34)
LRT test	0.079	0.669	0.135	0.129	0.478	<0.001

Adjust for: age(smooth), height(smooth), weight(smooth), BMI(smooth), overweight, history of cardiovascular and cerebrovascular diseases, history of diabetes, history of cancer, history of open surgery, history of hysteroscopy and laparoscopic surgery, age at menarche(smooth), menstrual regularity, amount of menses and dysmenorrhea.

Supplementary table 5. Baseline features of training and validation sets

Group	Test	Train	P-value
N	1044	2447	
EM			0.785
No	935 (89.56%)	2199 (89.87%)	
Yes	109 (10.44%)	248 (10.13%)	
Age, years	42.10 ± 9.44	41.94 ± 9.29	0.649
Height, cm	161.36 ± 4.74	161.20 ± 4.71	0.359
Weight, kg	62.44 ± 7.41	62.38 ± 7.24	0.826

BMI, kg/m^2	24.00 ± 2.83	24.03 ± 2.81	0.778
Overweight, n (%)			0.974
No	519 (49.71%)	1215 (49.65%)	
Yes	525 (50.29%)	1232 (50.35%)	
History of cardiovascular and cerebrovascular diseases, n (%)			0.037
No	977 (93.58%)	2332 (95.30%)	
Yes	67 (6.42%)	115 (4.70%)	
History of diabetes, n (%)			0.449
No	990 (94.83%)	2335 (95.42%)	
Yes	54 (5.17%)	112 (4.58%)	
History of cancer, n (%)			0.569
No	1032 (98.85%)	2413 (98.61%)	
Yes	12 (1.15%)	34 (1.39%)	
History of open surgery, n (%)			0.870
No	672 (64.37%)	1568 (64.08%)	
Yes	372 (35.63%)	879 (35.92%)	
History of hysteroscopy and laparoscopic surgery, n (%)			0.036
No	730 (69.92%)	1796 (73.40%)	
Yes	314 (30.08%)	651 (26.60%)	
Age at menarche, years	13.90 ± 1.34	13.93 ± 1.36	0.556
Menstrual regularity, n (%)			0.364
Erratical	252 (24.14%)	556 (22.72%)	
Rule	792 (75.86%)	1891 (77.28%)	
Amount of menses, n (%)			0.306
Less	41 (3.93%)	90 (3.68%)	
Normal	903 (86.49%)	2080 (85.00%)	
More	100 (9.58%)	277 (11.32%)	
Dysmenorrhea, n (%)			0.676
No	889 (85.15%)	2097 (85.70%)	
Yes	155 (14.85%)	350 (14.30%)	
HLD, mmol/L	1.56 ± 0.33	1.56 ± 0.33	0.979
LDL, mmol/L	2.72 ± 0.69	2.75 ± 0.69	0.380
VLDL, mmol/L	0.55 ± 0.20	0.55 ± 0.20	0.885
TC, mmol/L	4.84 ± 0.88	4.86 ± 0.88	0.475
TG, mmol/L	0.92 ± 0.58	0.94 ± 0.62	0.500
NHHR	2.18 ± 0.64	2.20 ± 0.67	0.383
LC, 10^9/L	1.65 ± 0.51	1.68 ± 0.52	0.184
NC, 10^9/L	2.66 ± 0.78	2.69 ± 0.80	0.419
PLT, 10^9/L	214.55 ± 48.64	215.90 ± 51.38	0.470
PLR	138.41 ± 42.08	136.33 ± 39.01	0.159
NLR	1.68 ± 0.47	1.67 ± 0.46	0.489
SII	350.12 ± 88.37	348.29 ± 86.92	0.570

Supplementary table 6. LASSO regression non zero coefficient

Variable	Coefficient
(Intercept)	-2.921049900
Age	-0.014831242
Height	.
Weight	-0.083836797
BMI	-0.069425270
Overweight	0.963371842
History of cardiovascular and cerebrovascular diseases	-0.727356250
History of diabetes	-0.044313724
History of cancer	-0.767545355
History of open surgery	.
History of hysteroscopy and laparoscopic surgery	-0.002724724
Age at menarche, years	-0.372449122
Menstrual regularity	0.581266704
Amount of menses	0.211797817
Dysmenorrhea	3.725224211
HDL	1.596018835
LDL	-0.899651628
VLDL	-0.692279533
TC	.
TG	-0.218198730
NHHR	1.188531876
LC	.
NC	-0.143936121
PLT	0.007252643
PLR	-0.013697926
NLR	-0.313508674
SII	0.016990908

Supplementary table 7. Training set logistic regression model

Variable	Estimate	Std. Error	z value	Pr(>|z|)
(Intercept)	-5.005862	3.833053	-1.306000	0.191562
Age	-0.020013	0.010629	-1.883000	0.059724
Weight	-0.077006	0.029712	-2.592000	0.009549
BMI	-0.068006	0.095730	-0.710000	0.477460
Overweight_Yes	0.633057	0.368754	1.717000	0.086026
History of cardiovascular and cerebrovascular diseases_Yes	-1.682519	0.738559	-2.278000	0.022720

History of diabetes_Yes	0.428980	0.427956	1.002000	0.316155
History of cancer_Yes	-1.411884	1.508621	-0.936000	0.349337
History of open surgery_Yes	0.167910	0.207327	0.810000	0.418008
History of hysteroscopy and laparoscopic surgery_Yes	0.006034	0.226049	0.027000	0.978704
Age_at_menarche11	0.638207	2.695811	0.237000	0.812858
Age_at_menarche12	1.224698	2.289239	0.535000	0.592663
Age_at_menarche13	1.436738	2.278759	0.630000	0.528373
Age_at_menarche14	1.520916	2.276479	0.668000	0.504070
Age_at_menarche15	1.694293	2.287253	0.741000	0.458842
Age_at_menarche16	-1.651363	2.290986	-0.721000	0.471027
Age_at_menarche17	-1.887815	2.320523	-0.814000	0.415914
Age_at_menarche18	-1.297095	2.419532	-0.536000	0.591894
Menstrual regularity_Yes	0.628753	0.281166	2.236000	0.025336
Amount of menses_Normal	0.901614	0.660192	1.366000	0.172038
Amount of menses_More	1.020625	0.709434	1.439000	0.150251
Dysmenorrhea_Yes	5.056011	0.351556	14.382000	0.000000
HDL	1.994092	0.765210	2.606000	0.009162
LDL	-1.317991	0.369627	-3.566000	0.000363
VLDL	-0.935338	0.679263	-1.377000	0.168516
TG	-0.400405	0.236298	-1.694000	0.090172
NHHR	1.634541	0.513489	3.183000	0.001457
NC	-0.306826	0.391973	-0.783000	0.433760
PLT	-0.000428	0.007533	-0.057000	0.954663
PLR	-0.019468	0.008735	-2.229000	0.025838
NLR	-1.808069	1.042652	-1.734000	0.082899
SII	0.027741	0.005556	4.993000	0.000001

Supplementary table 8. Hierarchical analysis

	N	EM<br>OR (95%CI) Pvalue
HDL		
Overweight		
No	1734	0.49 (0.29, 0.85) 0.0107
Yes	1757	0.35 (0.22, 0.58) <0.0001
History of cardiovascular and cerebrovascular diseases		
No	3309	0.39 (0.27, 0.56) <0.0001
Yes	182	0.19 (0.02, 1.89) 0.1558
History of diabetes		
No	3325	0.39 (0.27, 0.56) <0.0001
Yes	166	0.28 (0.03, 3.12) 0.3036
History of cancer		

No	3445	0.39 (0.27, 0.55) <0.0001
Yes	46	0.00 (0.00, Inf) 0.9999
History of open surgery		
No	2240	0.45 (0.29, 0.71) 0.0005
Yes	1251	0.29 (0.16, 0.53) <0.0001
History of hysteroscopy and laparoscopic surgery		
No	2526	0.43 (0.28, 0.65) <0.0001
Yes	965	0.27 (0.13, 0.56) 0.0004
Menstrual regularity		
Erratical	808	0.57 (0.23, 1.39) 0.2154
Rule	2683	0.37 (0.25, 0.54) <0.0001
Amount of menses		
Less	131	0.66 (0.07, 5.84) 0.7075
Normal	2983	0.40 (0.27, 0.59) <0.0001
More	377	0.27 (0.10, 0.72) 0.0087
Dysmenorrhea		
No	2986	0.44 (0.26, 0.74) 0.0021
Yes	505	0.18 (0.01, 4.01) 0.2797
LDL		
Overweight		
No	1734	0.82 (0.64, 1.06) 0.1318
Yes	1757	1.24 (1.01, 1.53) 0.0444
History of cardiovascular and cerebrovascular diseases		
No	3309	1.06 (0.90, 1.24) 0.4860
Yes	182	0.73 (0.26, 2.00) 0.5362
History of diabetes		
No	3325	1.05 (0.89, 1.23) 0.5825
Yes	166	1.15 (0.45, 2.91) 0.7737
History of cancer		
No	3445	1.05 (0.90, 1.23) 0.5482
Yes	46	0.00 (0.00, Inf) 0.9999
History of open surgery		
No	2240	1.13 (0.93, 1.38) 0.2079
Yes	1251	0.91 (0.69, 1.19) 0.4844
History of hysteroscopy and laparoscopic surgery		
No	2526	1.11 (0.93, 1.33) 0.2534
Yes	965	0.86 (0.62, 1.19) 0.3622
Menstrual regularity		
Erratical	808	1.50 (1.02, 2.22) 0.0418
Rule	2683	0.98 (0.83, 1.17) 0.8631
Amount of menses		

Less	131	0.79 (0.28, 2.21) 0.6521
Normal	2983	1.02 (0.86, 1.21) 0.8243
More	377	1.40 (0.92, 2.13) 0.1151
Dysmenorrhea		
No	2986	1.18 (0.94, 1.49) 0.1483
Yes	505	0.93 (0.30, 2.88) 0.9042
VLDL		
Overweight		
No	1734	1.37 (0.61, 3.09) 0.4408
Yes	1757	2.26 (1.12, 4.58) 0.0235
History of cardiovascular and cerebrovascular diseases		
No	3309	1.87 (1.10, 3.18) 0.0205
Yes	182	0.48 (0.02, 13.58) 0.6690
History of diabetes		
No	3325	1.74 (1.02, 2.97) 0.0437
Yes	166	5.00 (0.37, 67.57) 0.2260
History of cancer		
No	3445	1.85 (1.09, 3.12) 0.0224
Yes	46	0.00 (0.00, Inf) 0.9997
History of open surgery		
No	2240	1.44 (0.74, 2.79) 0.2874
Yes	1251	2.67 (1.14, 6.30) 0.0244
History of hysteroscopy and laparoscopic surgery		
No	2526	1.60 (0.86, 2.99) 0.1397
Yes	965	2.33 (0.88, 6.18) 0.0881
Menstrual regularity		
Erratical	808	3.69 (0.98, 13.96) 0.0540
Rule	2683	1.54 (0.87, 2.74) 0.1416
Amount of menses		
Less	131	0.15 (0.01, 4.00) 0.2538
Normal	2983	2.00 (1.12, 3.56) 0.0189
More	377	2.43 (0.62, 9.46) 0.1999
Dysmenorrhea		
No	2986	1.89 (0.87, 4.12) 0.1099
Yes	505	0.37 (0.01, 17.88) 0.6158
TC		
Overweight		
No	1734	0.82 (0.67, 1.00) 0.0477
Yes	1757	1.05 (0.88, 1.24) 0.6056
History of cardiovascular and cerebrovascular diseases		
No	3309	0.95 (0.83, 1.08) 0.3951

Yes	182	0.65 (0.29, 1.43) 0.2812
History of diabetes		
No	3325	0.93 (0.82, 1.06) 0.2897
Yes	166	1.07 (0.51, 2.22) 0.8585
History of cancer		
No	3445	0.94 (0.83, 1.07) 0.3276
Yes	46	0.00 (0.00, Inf) 0.9997
History of open surgery		
No	2240	0.99 (0.85, 1.16) 0.9431
Yes	1251	0.84 (0.67, 1.04) 0.1090
History of hysteroscopy and laparoscopic surgery		
No	2526	0.98 (0.85, 1.13) 0.7764
Yes	965	0.81 (0.63, 1.04) 0.0955
Menstrual regularity		
Erratical	808	1.28 (0.94, 1.75) 0.1180
Rule	2683	0.89 (0.77, 1.02) 0.0897
Amount of menses		
Less	131	0.69 (0.29, 1.65) 0.4061
Normal	2983	0.93 (0.81, 1.07) 0.3028
More	377	1.10 (0.78, 1.53) 0.5934
Dysmenorrhea		
No	2986	1.03 (0.86, 1.24) 0.7353
Yes	505	0.78 (0.34, 1.82) 0.5657
TG		
Overweight		
No	1734	1.15 (0.93, 1.42) 0.2095
Yes	1757	1.37 (1.08, 1.74) 0.0086
History of cardiovascular and cerebrovascular diseases		
No	3309	1.20 (1.03, 1.40) 0.0169
Yes	182	2.68 (0.92, 7.77) 0.0694
History of diabetes		
No	3325	1.22 (1.05, 1.42) 0.0084
Yes	166	0.98 (0.32, 2.97) 0.9725
History of cancer		
No	3445	1.23 (1.06, 1.43) 0.0068
Yes	46	0.00 (0.00, Inf) 0.9997
History of open surgery		
No	2240	1.09 (0.90, 1.32) 0.3882
Yes	1251	1.58 (1.22, 2.06) 0.0006
History of hysteroscopy and laparoscopic surgery		
No	2526	1.16 (0.98, 1.38) 0.0861

Yes	965	1.42 (1.05, 1.92) 0.0212
Menstrual regularity		
Erratical	808	1.07 (0.75, 1.53) 0.7154
Rule	2683	1.32 (1.10, 1.59) 0.0024
Amount of menses		
Less	131	0.61 (0.10, 3.65) 0.5898
Normal	2983	1.21 (1.03, 1.42) 0.0172
More	377	1.47 (0.93, 2.32) 0.1013
Dysmenorrhea		
No	2986	1.17 (0.94, 1.45) 0.1517
Yes	505	1.38 (0.28, 6.73) 0.6873
NHHR		
Overweight		
No	1734	1.25 (0.98, 1.58) 0.0680
Yes	1757	1.91 (1.53, 2.37) <0.0001
History of cardiovascular and cerebrovascular diseases		
No	3309	1.58 (1.36, 1.85) <0.0001
Yes	182	1.42 (0.47, 4.30) 0.5365
History of diabetes		
No	3325	1.58 (1.35, 1.84) <0.0001
Yes	166	1.87 (0.64, 5.52) 0.2555
History of cancer		
No	3445	1.59 (1.36, 1.85) <0.0001
Yes	46	7.72 (0.00, Inf) 1.0000
History of open surgery		
No	2240	1.58 (1.31, 1.92) <0.0001
Yes	1251	1.58 (1.23, 2.03) 0.0004
History of hysteroscopy and laparoscopic surgery		
No	2526	1.61 (1.35, 1.93) <0.0001
Yes	965	1.50 (1.12, 2.00) 0.0072
Menstrual regularity		
Erratical	808	1.88 (1.27, 2.80) 0.0017
Rule	2683	1.53 (1.30, 1.81) <0.0001
Amount of menses		
Less	131	0.98 (0.34, 2.79) 0.9650
Normal	2983	1.53 (1.30, 1.81) <0.0001
More	377	2.30 (1.50, 3.53) 0.0001
Dysmenorrhea		
No	2986	1.70 (1.36, 2.11) <0.0001
Yes	505	1.40 (0.44, 4.45) 0.5703

Adjust for: age(smooth), height(smooth), weight(smooth), BMI(smooth) and age at

menarche(smooth).

Supplementary table 9. Subgroup analysis of HDL

Variables: HDL	n (%)	Univariate analysis			Multivariate analysis		
		OR (95%CI)	*P*	P for interaction	OR (95%CI)	*P*	P for interaction
All patients	3491 (100.00)	0.39 (0.27, 0.56)	<0.001		0.39 (0.27, 0.55)	<0.001	
Overweight				0.524			0.546
No	1734 (49.67)	0.45 (0.26, 0.77)	0.003		0.49 (0.29, 0.85)	0.011	
Yes	1757 (50.33)	0.36 (0.22, 0.58)	<0.001		0.35 (0.22, 0.58)	<0.001	
History of cardiovascular and cerebrovascular diseases				0.542			0.505
No	3309 (94.79)	0.40 (0.28, 0.57)	<0.001		0.39 (0.27, 0.56)	<0.001	
Yes	182 (5.21)	0.19 (0.02, 2.09)	0.175		0.19 (0.02, 1.89)	0.156	
History of diabetes				0.658			0.744
No	3325 (95.24)	0.40 (0.28, 0.57)	<0.001		0.39 (0.27, 0.56)	<0.001	
Yes	166 (4.76)	0.24 (0.03, 2.25)	0.212		0.28 (0.03, 3.12)	0.304	
History of cancer				0.792			0.827
No	3445 (98.68)	0.39 (0.27, 0.56)	<0.001		0.39 (0.27, 0.55)	<0.001	
Yes	46 (1.32)	0.66 (0.01,	0.838		0.00 (0.00,	1.000	

		33.29)			Inf)		
History of open surgery				0.191			0.243
No	2240 (64.16)	0.47 (0.30, 0.73)	<0.001		0.45 (0.29, 0.71)	<0.001	
Yes	1251 (35.84)	0.29 (0.16, 0.52)	<0.001		0.29 (0.16, 0.53)	<0.001	
History of hysteroscopy and laparoscopic surgery				0.251			0.316
No	2526 (72.36)	0.44 (0.29, 0.67)	<0.001		0.43 (0.28, 0.65)	<0.001	
Yes	965 (27.64)	0.27 (0.13, 0.56)	<0.001		0.27 (0.13, 0.56)	<0.001	
Menstrual regularity				0.536			0.531
Erratical	808 (23.15)	0.51 (0.21, 1.23)	0.132		0.57 (0.23, 1.39)	0.215	
Rule	2683 (76.85)	0.38 (0.25, 0.56)	<0.001		0.37 (0.25, 0.54)	<0.001	
Amount of menses				0.716			0.629
Less	131 (3.75)	0.58 (0.08, 4.01)	0.577		0.66 (0.07, 5.84)	0.708	
Normal	377 (10.80)	0.28 (0.11, 0.73)	0.010		0.27 (0.10, 0.72)	0.009	
More	2983 (85.45)	0.41 (0.28, 0.60)	<0.001		0.40 (0.27, 0.59)	<0.001	
Dysmenorrhea				0.803			0.813
No	2986 (85.53)	0.45 (0.27, 0.76)	0.003		0.44 (0.26, 0.74)	0.002	
Yes	505	0.41	0.002		0.18	0.280	

	(14.47)	(0.23, 0.72)			(0.01, 4.01)		

Adjust for: age(smooth), height(smooth), weight(smooth), BMI(smooth) and age at menarche(smooth).

Supplementary table 10. Subgroup analysis of LDL

Variables: LDL	n (%)	Univariate analysis			Multivariate analysis		
		OR (95%CI)	*P*	P for interaction	OR (95%CI)	*P*	P for interaction
All patients	3491 (100.00)	1.06 (0.90, 1.24)	0.501		0.39 (0.27, 0.55)	<0.001	
Overweight				0.010			0.546
No	1734 (49.67)	0.83 (0.65, 1.06)	0.140		0.49 (0.29, 0.85)	0.011	
Yes	1757 (50.33)	1.27 (1.03, 1.56)	0.025		0.35 (0.22, 0.58)	<0.001	
History of cardiovascular and cerebrovascular diseases				0.461			0.505
No	3309 (94.79)	1.06 (0.91, 1.25)	0.454		0.39 (0.27, 0.56)	<0.001	
Yes	182 (5.21)	0.73 (0.27, 1.99)	0.538		0.19 (0.02, 1.89)	0.156	
History of diabetes				0.718			0.744
No	3325 (95.24)	1.05 (0.89, 1.23)	0.552		0.39 (0.27, 0.56)	<0.001	
Yes	166 (4.76)	1.25 (0.50, 3.12)	0.637		0.28 (0.03, 3.12)	0.304	
History of cancer				0.539			0.827
No	3445	1.05	0.53		0.39	<0.00	

	(98.68)	(0.90, 1.23)	0		(0.27, 0.55)	1	
Yes	46 (1.32)	1.83 (0.32, 10.48)	0.496		0.00 (0.00, Inf)	1.000	
History of open surgery				0.148			0.243
No	2240 (64.16)	1.15 (0.95, 1.40)	0.159		0.45 (0.29, 0.71)	<0.001	
Yes	1251 (35.84)	0.90 (0.69, 1.18)	0.446		0.29 (0.16, 0.53)	<0.001	
History of hysteroscopy and laparoscopic surgery				0.138			0.316
No	2526 (72.36)	1.13 (0.94, 1.35)	0.184		0.43 (0.28, 0.65)	<0.001	
Yes	965 (27.64)	0.86 (0.62, 1.18)	0.347		0.27 (0.13, 0.56)	<0.001	
Menstrual regularity				0.092			0.531
Erratical	808 (23.15)	1.42 (0.98, 2.07)	0.065		0.57 (0.23, 1.39)	0.215	
Rule	2683 (76.85)	0.99 (0.84, 1.18)	0.953		0.37 (0.25, 0.54)	<0.001	
Amount of menses				0.328			0.629
Less	131 (3.75)	0.86 (0.33, 2.24)	0.753		0.66 (0.07, 5.84)	0.708	
Normal	377 (10.80)	1.42 (0.93, 2.16)	0.100		0.27 (0.10, 0.72)	0.009	
More	2983 (85.45)	1.02 (0.86, 1.22)	0.812		0.40 (0.27, 0.59)	<0.001	
Dysmenorrhea				0.364			0.813

No	2986 (85.53)	1.18 (0.94, 1.48)	0.153		0.44 (0.26, 0.74)	0.002	
Yes	505 (14.47)	1.01 (0.78, 1.30)	0.967		0.18 (0.01, 4.01)	0.280	

Adjust for: age(smooth), height(smooth), weight(smooth), BMI(smooth) and age at menarche(smooth).

Supplementary table 11. Subgroup analysis of VLDL

Variables: VLDL	n (%)	Univariate analysis			Multivariate analysis		
		OR (95%CI)	*P*	P for interaction	OR (95%CI)	*P*	P for interaction
All patients	3491 (100.00)	1.81 (1.08, 3.05)	0.025		0.39 (0.27, 0.55)	<0.001	
Overweight				0.393			0.546
No	1734 (49.67)	1.40 (0.62, 3.13)	0.417		0.49 (0.29, 0.85)	0.011	
Yes	1757 (50.33)	2.21 (1.12, 4.38)	0.023		0.35 (0.22, 0.58)	<0.001	
History of cardiovascular and cerebrovascular diseases				0.563			0.505
No	3309 (94.79)	1.86 (1.10, 3.15)	0.020		0.39 (0.27, 0.56)	<0.001	
Yes	182 (5.21)	0.67 (0.02, 22.38)	0.826		0.19 (0.02, 1.89)	0.156	
History of diabetes				0.511			0.744
No	3325 (95.24)	1.75 (1.03, 2.98)	0.039		0.39 (0.27, 0.56)	<0.001	
Yes	166 (4.76)	4.07 (0.37,	0.251		0.28 (0.03,	0.304	

		44.72)			3.12)		
History of cancer				0.887			0.827
No	3445 (98.68)	1.84 (1.09, 3.09)	0.023		0.39 (0.27, 0.55)	<0.001	
Yes	46 (1.32)	1.17 (0.00, 628.16)	0.960		0.00 (0.00, Inf)	1.000	
History of open surgery				0.316			0.243
No	2240 (64.16)	1.48 (0.77, 2.86)	0.241		0.45 (0.29, 0.71)	<0.001	
Yes	1251 (35.84)	2.57 (1.10, 6.01)	0.029		0.29 (0.16, 0.53)	<0.001	
History of hysteroscopy and laparoscopic surgery				0.566			0.316
No	2526 (72.36)	1.65 (0.89, 3.06)	0.114		0.43 (0.28, 0.65)	<0.001	
Yes	965 (27.64)	2.30 (0.89, 5.98)	0.087		0.27 (0.13, 0.56)	<0.001	
Menstrual regularity				0.420			0.531
Erratical	808 (23.15)	2.87 (0.77, 10.67)	0.116		0.57 (0.23, 1.39)	0.215	
Rule	2683 (76.85)	1.58 (0.90, 2.80)	0.114		0.37 (0.25, 0.54)	<0.001	
Amount of menses				0.030			0.629
Less	131 (3.75)	0.04 (0.01, 0.91)	0.043		0.66 (0.07, 5.84)	0.708	
Normal	377 (10.80)	2.49 (0.65, 9.58)	0.184		0.27 (0.10, 0.72)	0.009	

More	2983 (85.45)	2.03 (1.14, 3.60)	0.016		0.40 (0.27, 0.59)	<0.001	
Dysmenorrhea				0.686			0.813
No	2986 (85.53)	1.96 (0.91, 4.22)	0.085		0.44 (0.26, 0.74)	0.002	
Yes	505 (14.47)	1.55 (0.68, 3.55)	0.295		0.18 (0.01, 4.01)	0.280	

Adjust for: age(smooth), height(smooth), weight(smooth), BMI(smooth) and age at menarche(smooth).

Supplementary table 12. Subgroup analysis of TC

		Univariate analysis			Multivariate analysis		
Variables: TC	n (%)	OR (95%CI)	*P*	P for interaction	OR (95%CI)	*P*	P for interaction
All patients	3491 (100.00)	0.94 (0.83, 1.07)	0.364		0.39 (0.27, 0.55)	<0.001	
Overweight				0.039			0.546
No	1734 (49.67)	0.81 (0.67, 0.99)	0.035		0.49 (0.29, 0.85)	0.011	
Yes	1757 (50.33)	1.06 (0.90, 1.25)	0.489		0.35 (0.22, 0.58)	<0.001	
History of cardiovascular and cerebrovascular diseases				0.369			0.505
No	3309 (94.79)	0.95 (0.84, 1.08)	0.437		0.39 (0.27, 0.56)	<0.001	
Yes	182 (5.21)	0.66 (0.30, 1.47)	0.311		0.19 (0.02, 1.89)	0.156	
History of diabetes				0.706			0.744
No	3325	0.94	0.33		0.39	<0.00	

	(95.24)	(0.83, 1.07)	4			(0.27, 0.56)	1	
Yes	166 (4.76)	1.09 (0.52, 2.26)	0.827			0.28 (0.03, 3.12)	0.304	
History of cancer				0.589				0.827
No	3445 (98.68)	0.94 (0.83, 1.07)	0.353			0.39 (0.27, 0.55)	<0.001	
Yes	46 (1.32)	1.38 (0.35, 5.53)	0.648			0.00 (0.00, Inf)	1.000	
History of open surgery				0.147				0.243
No	2240 (64.16)	1.01 (0.86, 1.18)	0.897			0.45 (0.29, 0.71)	<0.001	
Yes	1251 (35.84)	0.83 (0.67, 1.03)	0.091			0.29 (0.16, 0.53)	<0.001	
History of hysteroscopy and laparoscopic surgery				0.154				0.316
No	2526 (72.36)	1.00 (0.86, 1.15)	0.949			0.43 (0.28, 0.65)	<0.001	
Yes	965 (27.64)	0.81 (0.63, 1.04)	0.094			0.27 (0.13, 0.56)	<0.001	
Menstrual regularity				0.077				0.531
Erratical	808 (23.15)	1.22 (0.90, 1.65)	0.209			0.57 (0.23, 1.39)	0.215	
Rule	2683 (76.85)	0.90 (0.78, 1.03)	0.123			0.37 (0.25, 0.54)	<0.001	
Amount of menses				0.416				0.629
Less	131 (3.75)	0.66 (0.30,	0.295			0.66 (0.07,	0.708	

		1.44)			5.84)		
Normal	377 (10.80)	1.11 (0.79, 1.55)	0.544		0.27 (0.10, 0.72)	0.009	
More	2983 (85.45)	0.93 (0.81, 1.07)	0.340		0.40 (0.27, 0.59)	<0.001	
Dysmenorrhea				0.354			0.813
No	2986 (85.53)	1.04 (0.86, 1.24)	0.702		0.44 (0.26, 0.74)	0.002	
Yes	505 (14.47)	0.91 (0.74, 1.12)	0.369		0.18 (0.01, 4.01)	0.280	

Adjust for: age(smooth), height(smooth), weight(smooth), BMI(smooth) and age at menarche(smooth).

Supplementary table 13. Subgroup analysis of TG

Variables: TG	n (%)	Univariate analysis			Multivariate analysis		
		OR (95%CI)	*P*	P for interaction	OR (95%CI)	*P*	P for interaction
All patients	3491 (100.00)	1.21 (1.05, 1.41)	0.010		0.39 (0.27, 0.55)	<0.001	
Overweight				0.225			0.546
No	1734 (49.67)	1.12 (0.92, 1.38)	0.261		0.49 (0.29, 0.85)	0.011	
Yes	1757 (50.33)	1.35 (1.08, 1.70)	0.008		0.35 (0.22, 0.58)	<0.001	
History of cardiovascular and cerebrovascular diseases				0.134			0.505
No	3309 (94.79)	1.19 (1.03, 1.39)	0.020		0.39 (0.27, 0.56)	<0.001	
Yes	182 (5.21)	2.74 (0.98,	0.054		0.19 (0.02,	0.156	

		7.65)			1.89)		
History of diabetes				0.810			0.744
No	3325 (95.24)	1.22 (1.05, 1.42)	0.010		0.39 (0.27, 0.56)	<0.001	
Yes	166 (4.76)	1.08 (0.41, 2.88)	0.874		0.28 (0.03, 3.12)	0.304	
History of cancer				0.456			0.827
No	3445 (98.68)	1.22 (1.05, 1.42)	0.009		0.39 (0.27, 0.55)	<0.001	
Yes	46 (1.32)	0.40 (0.01, 11.86)	0.595		0.00 (0.00, Inf)	1.000	
History of open surgery				0.033			0.243
No	2240 (64.16)	1.09 (0.90, 1.32)	0.358		0.45 (0.29, 0.71)	<0.001	
Yes	1251 (35.84)	1.55 (1.20, 2.01)	<0.001		0.29 (0.16, 0.53)	<0.001	
History of hysteroscopy and laparoscopic surgery				0.297			0.316
No	2526 (72.36)	1.16 (0.98, 1.38)	0.079		0.43 (0.28, 0.65)	<0.001	
Yes	965 (27.64)	1.40 (1.04, 1.88)	0.026		0.27 (0.13, 0.56)	<0.001	
Menstrual regularity				0.138			0.531
Erratical	808 (23.15)	1.03 (0.73, 1.45)	0.863		0.57 (0.23, 1.39)	0.215	
Rule	2683 (76.85)	1.33 (1.11, 1.60)	0.002		0.37 (0.25, 0.54)	<0.001	

Amount of menses				0.337			0.629
Less	131 (3.75)	0.49 (0.09, 2.58)	0.398		0.66 (0.07, 5.84)	0.708	
Normal	377 (10.80)	1.50 (0.95, 2.36)	0.080		0.27 (0.10, 0.72)	0.009	
More	2983 (85.45)	1.20 (1.03, 1.41)	0.023		0.40 (0.27, 0.59)	<0.001	
Dysmenorrhea				0.528			0.813
No	2986 (85.53)	1.17 (0.95, 1.42)	0.134		0.44 (0.26, 0.74)	0.002	
Yes	505 (14.47)	1.31 (0.97, 1.76)	0.077		0.18 (0.01, 4.01)	0.280	

Adjust for: age(smooth), height(smooth), weight(smooth), BMI(smooth) and age at menarche(smooth).

Supplementary table 14. Subgroup analysis of NHHR

		Univariate analysis			Multivariate analysis		
Variables: NHHR	n (%)	OR (95%CI)	*P*	P for interaction	OR (95%CI)	*P*	P for interaction
All patients	3491 (100.00)	1.57 (1.35, 1.82)	<0.001		1.59 (1.36, 1.85)	<0.001	
Overweight				0.013			0.011
No	1734 (49.67)	1.28 (1.02, 1.61)	0.036		1.25 (0.98, 1.58)	0.068	
Yes	1757 (50.33)	1.88 (1.53, 2.32)	<0.001		1.91 (1.53, 2.37)	<0.001	
History of cardiovascular and cerebrovascular diseases				0.790			0.825
No	3309	1.57	<0.00		1.58	<0.00	

	(94.79)	(1.35, 1.83)	1		(1.36, 1.85)	1	
Yes	182 (5.21)	1.36 (0.47, 3.89)	0.568		1.42 (0.47, 4.30)	0.537	
History of diabetes				0.744			0.826
No	3325 (95.24)	1.56 (1.34, 1.82)	<0.001		1.58 (1.35, 1.84)	<0.001	
Yes	166 (4.76)	1.83 (0.72, 4.68)	0.207		1.87 (0.64, 5.52)	0.256	
History of cancer				0.915			0.899
No	3445 (98.68)	1.57 (1.35, 1.83)	<0.001		1.59 (1.36, 1.85)	<0.001	
Yes	46 (1.32)	1.43 (0.27, 7.66)	0.673		7.72 (0.00, Inf)	1.000	
History of open surgery				0.985			0.983
No	2240 (64.16)	1.57 (1.30, 1.90)	<0.001		1.58 (1.31, 1.92)	<0.001	
Yes	1251 (35.84)	1.57 (1.22, 2.01)	<0.001		1.58 (1.23, 2.03)	<0.001	
History of hysteroscopy and laparoscopic surgery				0.631			0.734
No	2526 (72.36)	1.60 (1.35, 1.91)	<0.001		1.61 (1.35, 1.93)	<0.001	
Yes	965 (27.64)	1.48 (1.10, 1.97)	0.009		1.50 (1.12, 2.00)	0.007	
Menstrual regularity				0.469			0.417
Erratical	808 (23.15)	1.78 (1.22,	0.003		1.88 (1.27,	0.002	

		2.58)			2.80)		
Rule	2683 (76.85)	1.53 (1.29, 1.80)	<0.001		1.53 (1.30, 1.81)	<0.001	
Amount of menses				0.118			0.113
Less	131 (3.75)	0.99 (0.38, 2.57)	0.990		0.98 (0.34, 2.79)	0.965	
Normal	377 (10.80)	2.29 (1.50, 3.49)	<0.001		2.30 (1.50, 3.53)	<0.001	
More	2983 (85.45)	1.51 (1.28, 1.78)	<0.001		1.53 (1.30, 1.81)	<0.001	
Dysmenorrhea				0.490			0.285
No	2986 (85.53)	1.65 (1.33, 2.04)	<0.001		1.70 (1.36, 2.11)	<0.001	
Yes	505 (14.47)	1.47 (1.13, 1.90)	0.004		1.40 (0.44, 4.45)	0.570	

Adjust for: age(smooth), height(smooth), weight(smooth), BMI(smooth) and age at menarche(smooth).

Figure

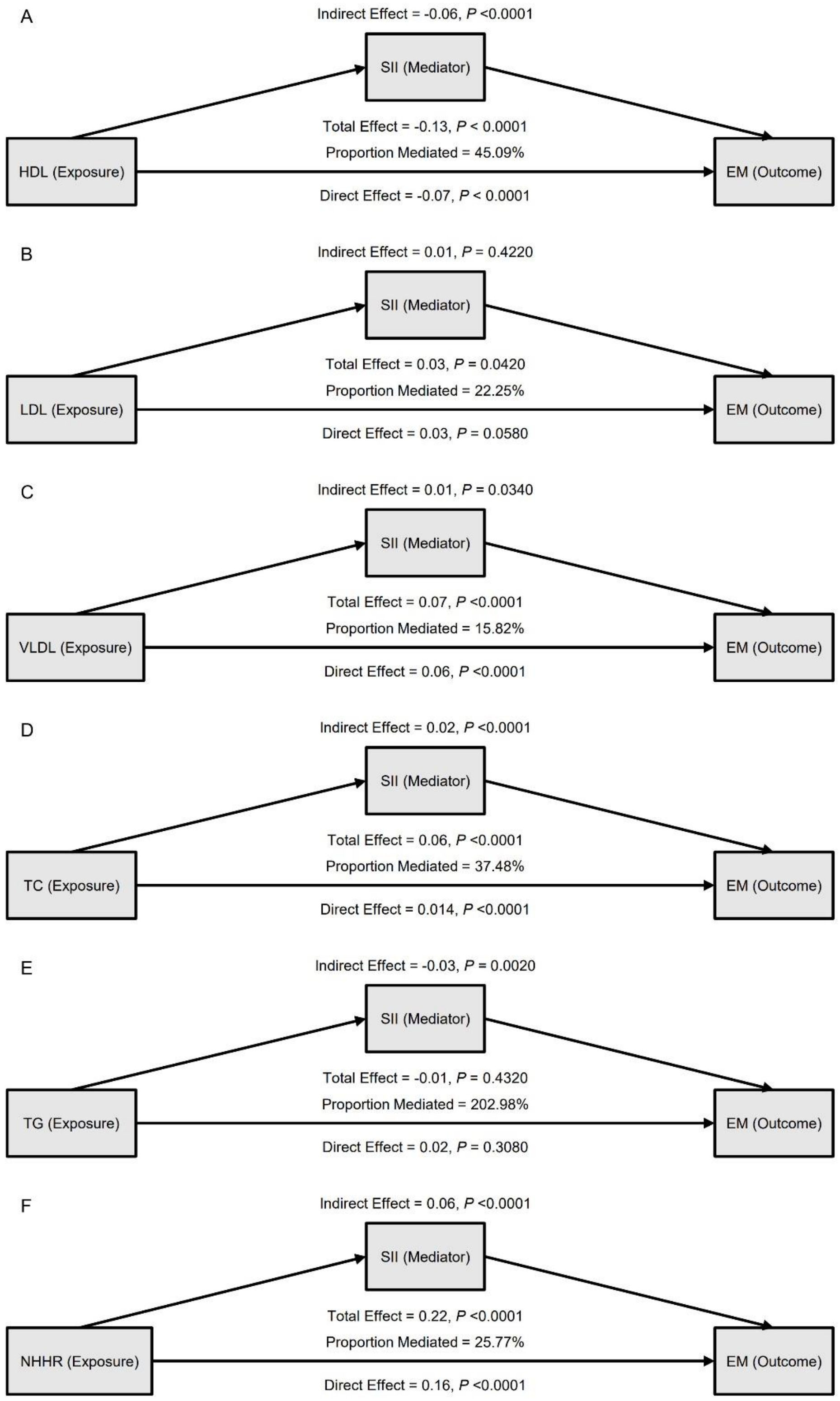

Supplementary Figure 1. Analysis of the mediating role of inflammation between blood lipids and EM in external validation

Variables	n (%)	OR (95%CI)	P	P for interaction
All patients	3491 (100.00)	0.39 (0.27 ~ 0.56)	<.001	
Overweight				0.524
No	1734 (49.67)	0.45 (0.26 ~ 0.77)	0.003	
Yes	1757 (50.33)	0.36 (0.22 ~ 0.58)	<.001	
Cardiovascular and cerebrovascular diseases				0.542
No	3309 (94.79)	0.40 (0.28 ~ 0.57)	<.001	
Yes	182 (5.21)	0.19 (0.02 ~ 2.09)	0.175	
DM				0.658
No	3325 (95.24)	0.40 (0.28 ~ 0.57)	<.001	
Yes	166 (4.76)	0.24 (0.03 ~ 2.25)	0.212	
CA				0.792
No	3445 (98.68)	0.39 (0.27 ~ 0.56)	<.001	
Yes	46 (1.32)	0.66 (0.01 ~ 33.29)	0.838	
History of abdominal surgery				0.191
No	2240 (64.16)	0.47 (0.30 ~ 0.73)	<.001	
Yes	1251 (35.84)	0.29 (0.16 ~ 0.52)	<.001	
History of cesarean section				0.251
No	2526 (72.36)	0.44 (0.29 ~ 0.67)	<.001	
Yes	965 (27.64)	0.27 (0.13 ~ 0.56)	<.001	
Menstrual regularity				0.536
No	808 (23.15)	0.51 (0.21 ~ 1.23)	0.132	
Yes	2683 (76.85)	0.38 (0.25 ~ 0.56)	<.001	
Amount of menses				0.716
Less	131 (3.75)	0.58 (0.08 ~ 4.01)	0.577	
More	377 (10.80)	0.28 (0.11 ~ 0.73)	0.010	
Normal	2983 (85.45)	0.41 (0.28 ~ 0.60)	<.001	
Dysmenorrhea				0.803
No	2986 (85.53)	0.45 (0.27 ~ 0.76)	0.003	
Yes	505 (14.47)	0.41 (0.23 ~ 0.72)	0.002	

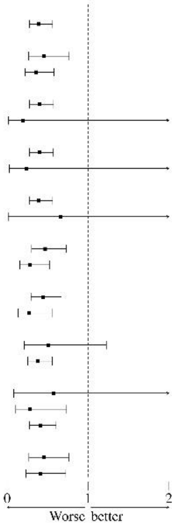

Supplementary table 2. Forest plot of HDL univariate analysis

Adjust for: age(smooth), height(smooth), weight(smooth), BMI(smooth) and age at menarche(smooth).

Variables	n (%)	OR (95%CI)	P	P for interaction
All patients	3491 (100.00)	0.39 (0.27 ~ 0.55)	<.001	
Overweight				0.546
No	1734 (49.67)	0.49 (0.29 ~ 0.85)	0.011	
Yes	1757 (50.33)	0.35 (0.22 ~ 0.58)	<.001	
Cardiovascular and cerebrovascular diseases				0.505
No	3309 (94.79)	0.39 (0.27 ~ 0.56)	<.001	
Yes	182 (5.21)	0.19 (0.02 ~ 1.89)	0.156	
DM				0.744
No	3325 (95.24)	0.39 (0.27 ~ 0.56)	<.001	
Yes	166 (4.76)	0.28 (0.03 ~ 3.12)	0.304	
CA				0.827
No	3445 (98.68)	0.39 (0.27 ~ 0.55)	<.001	
Yes	46 (1.32)	0.00 (0.00 ~ Inf)	1.000	
History of abdominal surgery				0.243
No	2240 (64.16)	0.45 (0.29 ~ 0.71)	<.001	
Yes	1251 (35.84)	0.29 (0.16 ~ 0.53)	<.001	
History of cesarean section				0.316
No	2526 (72.36)	0.43 (0.28 ~ 0.65)	<.001	
Yes	965 (27.64)	0.27 (0.13 ~ 0.56)	<.001	
Menstrual regularity				0.531
No	808 (23.15)	0.57 (0.23 ~ 1.39)	0.215	
Yes	2683 (76.85)	0.37 (0.25 ~ 0.54)	<.001	
Amount of menses				0.629
Less	131 (3.75)	0.66 (0.07 ~ 5.84)	0.708	
More	377 (10.80)	0.27 (0.10 ~ 0.72)	0.009	
Normal	2983 (85.45)	0.40 (0.27 ~ 0.59)	<.001	
Dysmenorrhea				0.813
No	2986 (85.53)	0.44 (0.26 ~ 0.74)	0.002	
Yes	505 (14.47)	0.18 (0.01 ~ 4.01)	0.280	

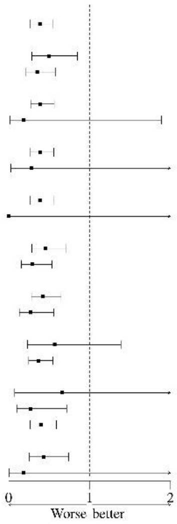

Supplementary table 3. Forest plot of HDL multifactor analysis

Adjust for: age(smooth), height(smooth), weight(smooth), BMI(smooth) and age at menarche(smooth).

Variables	n (%)	OR (95%CI)	P	P for interaction
All patients	3491 (100.00)	1.06 (0.90 ~ 1.24)	0.501	
Overweight				0.010
No	1734 (49.67)	0.83 (0.65 ~ 1.06)	0.140	
Yes	1757 (50.33)	1.27 (1.03 ~ 1.56)	0.025	
Cardiovascular and cerebrovascular diseases				0.461
No	3309 (94.79)	1.06 (0.91 ~ 1.25)	0.454	
Yes	182 (5.21)	0.73 (0.27 ~ 1.99)	0.538	
DM				0.718
No	3325 (95.24)	1.05 (0.89 ~ 1.23)	0.552	
Yes	166 (4.76)	1.25 (0.50 ~ 3.12)	0.637	
CA				0.539
No	3445 (98.68)	1.05 (0.90 ~ 1.23)	0.530	
Yes	46 (1.32)	1.83 (0.32 ~ 10.48)	0.496	
History of abdominal surgery				0.148
No	2240 (64.16)	1.15 (0.95 ~ 1.40)	0.159	
Yes	1251 (35.84)	0.90 (0.69 ~ 1.18)	0.446	
History of cesarean section				0.138
No	2526 (72.36)	1.13 (0.94 ~ 1.35)	0.184	
Yes	965 (27.64)	0.86 (0.62 ~ 1.18)	0.347	
Menstrual regularity				0.092
No	808 (23.15)	1.42 (0.98 ~ 2.07)	0.065	
Yes	2683 (76.85)	0.99 (0.84 ~ 1.18)	0.953	
Amount of menses				0.328
Less	131 (3.75)	0.86 (0.33 ~ 2.24)	0.753	
More	377 (10.80)	1.42 (0.93 ~ 2.16)	0.100	
Normal	2983 (85.45)	1.02 (0.86 ~ 1.22)	0.812	
Dysmenorrhea				0.364
No	2986 (85.53)	1.18 (0.94 ~ 1.48)	0.153	
Yes	505 (14.47)	1.01 (0.78 ~ 1.30)	0.967	

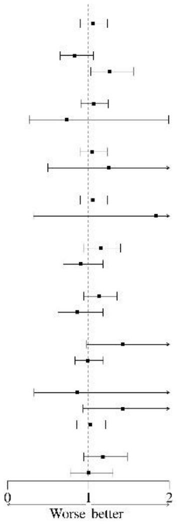

Supplementary table 4. Forest plot of LDL univariate analysis

Adjust for: age(smooth), height(smooth), weight(smooth), BMI(smooth) and age at menarche(smooth).

Variables	n (%)	OR (95%CI)	P	P for interaction
All patients	3491 (100.00)	1.05 (0.90 ~ 1.23)	0.535	
Overweight				0.010
No	1734 (49.67)	0.82 (0.64 ~ 1.06)	0.132	
Yes	1757 (50.33)	1.24 (1.01 ~ 1.53)	0.044	
Cardiovascular and cerebrovascular diseases				0.473
No	3309 (94.79)	1.06 (0.90 ~ 1.24)	0.486	
Yes	182 (5.21)	0.73 (0.26 ~ 2.00)	0.536	
DM				0.760
No	3325 (95.24)	1.05 (0.89 ~ 1.23)	0.582	
Yes	166 (4.76)	1.15 (0.45 ~ 2.91)	0.774	
CA				0.623
No	3445 (98.68)	1.05 (0.90 ~ 1.23)	0.548	
Yes	46 (1.32)	0.00 (0.00 ~ Inf)	1.000	
History of abdominal surgery				0.197
No	2240 (64.16)	1.13 (0.93 ~ 1.38)	0.208	
Yes	1251 (35.84)	0.91 (0.69 ~ 1.19)	0.484	
History of cesarean section				0.213
No	2526 (72.36)	1.11 (0.93 ~ 1.33)	0.253	
Yes	965 (27.64)	0.86 (0.62 ~ 1.19)	0.362	
Menstrual regularity				0.081
No	808 (23.15)	1.50 (1.02 ~ 2.22)	0.042	
Yes	2683 (76.85)	0.98 (0.83 ~ 1.17)	0.863	
Amount of menses				0.357
Less	131 (3.75)	0.79 (0.28 ~ 2.21)	0.652	
More	377 (10.80)	1.40 (0.92 ~ 2.13)	0.115	
Normal	2983 (85.45)	1.02 (0.86 ~ 1.21)	0.824	
Dysmenorrhea				0.441
No	2986 (85.53)	1.18 (0.94 ~ 1.49)	0.148	
Yes	505 (14.47)	0.93 (0.30 ~ 2.88)	0.904	

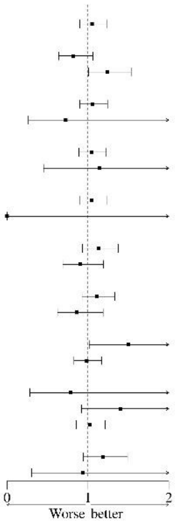

Supplementary table 5. Forest plot of LDL multifactor analysis

Adjust for: age(smooth), height(smooth), weight(smooth), BMI(smooth) and age at menarche(smooth).

Variables	n (%)	OR (95%CI)	P	P for interaction
All patients	3491 (100.00)	1.81 (1.08 ~ 3.05)	0.025	
Overweight				0.393
No	1734 (49.67)	1.40 (0.62 ~ 3.13)	0.417	
Yes	1757 (50.33)	2.21 (1.12 ~ 4.38)	0.023	
Cardiovascular and cerebrovascular diseases				0.563
No	3309 (94.79)	1.86 (1.10 ~ 3.15)	0.020	
Yes	182 (5.21)	0.67 (0.02 ~ 22.38)	0.826	
DM				0.511
No	3325 (95.24)	1.75 (1.03 ~ 2.98)	0.039	
Yes	166 (4.76)	4.07 (0.37 ~ 44.72)	0.251	
CA				0.887
No	3445 (98.68)	1.84 (1.09 ~ 3.09)	0.023	
Yes	46 (1.32)	1.17 (0.00 ~ 628.16)	0.960	
History of abdominal surgery				0.316
No	2240 (64.16)	1.48 (0.77 ~ 2.86)	0.241	
Yes	1251 (35.84)	2.57 (1.10 ~ 6.01)	0.029	
History of cesarean section				0.566
No	2526 (72.36)	1.65 (0.89 ~ 3.06)	0.114	
Yes	965 (27.64)	2.30 (0.89 ~ 5.98)	0.087	
Menstrual regularity				0.420
No	808 (23.15)	2.87 (0.77 ~ 10.67)	0.116	
Yes	2683 (76.85)	1.58 (0.90 ~ 2.80)	0.114	
Amount of menses				0.030
Less	131 (3.75)	0.04 (0.01 ~ 0.91)	0.043	
More	377 (10.80)	2.49 (0.65 ~ 9.58)	0.184	
Normal	2983 (85.45)	2.03 (1.14 ~ 3.60)	0.016	
Dysmenorrhea				0.686
No	2986 (85.53)	1.96 (0.91 ~ 4.22)	0.085	
Yes	505 (14.47)	1.55 (0.68 ~ 3.55)	0.295	

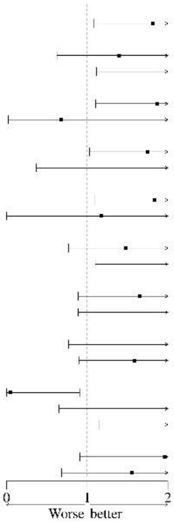

Supplementary table 6. Forest Map of VLDL Single Factor Analysis

Adjust for: age(smooth), height(smooth), weight(smooth), BMI(smooth) and age at menarche(smooth).

Variables	n (%)	OR (95%CI)	P	P for interaction
All patients	3491 (100.00)	1.81 (1.07 ~ 3.06)	0.026	
Overweight				0.395
No	1734 (49.67)	1.37 (0.61 ~ 3.09)	0.441	
Yes	1757 (50.33)	2.26 (1.12 ~ 4.58)	0.024	
Cardiovascular and cerebrovascular diseases				0.508
No	3309 (94.79)	1.87 (1.10 ~ 3.18)	0.021	
Yes	182 (5.21)	0.48 (0.02 ~ 13.58)	0.669	
DM				0.467
No	3325 (95.24)	1.74 (1.02 ~ 2.97)	0.044	
Yes	166 (4.76)	5.00 (0.37 ~ 67.57)	0.226	
CA				0.761
No	3445 (98.68)	1.85 (1.09 ~ 3.12)	0.022	
Yes	46 (1.32)	0.00 (0.00 ~ Inf)	1.000	
History of abdominal surgery				0.263
No	2240 (64.16)	1.44 (0.74 ~ 2.79)	0.287	
Yes	1251 (35.84)	2.67 (1.14 ~ 6.30)	0.024	
History of cesarean section				0.463
No	2526 (72.36)	1.60 (0.86 ~ 2.99)	0.140	
Yes	965 (27.64)	2.33 (0.88 ~ 6.18)	0.088	
Menstrual regularity				0.360
No	808 (23.15)	3.69 (0.98 ~ 13.96)	0.054	
Yes	2683 (76.85)	1.54 (0.87 ~ 2.74)	0.142	
Amount of menses				0.067
Less	131 (3.75)	0.15 (0.01 ~ 4.00)	0.254	
More	377 (10.80)	2.43 (0.63 ~ 9.46)	0.200	
Normal	2983 (85.45)	2.00 (1.12 ~ 3.56)	0.019	
Dysmenorrhea				0.846
No	2986 (85.53)	1.89 (0.87 ~ 4.12)	0.110	
Yes	505 (14.47)	0.37 (0.01 ~ 17.88)	0.616	

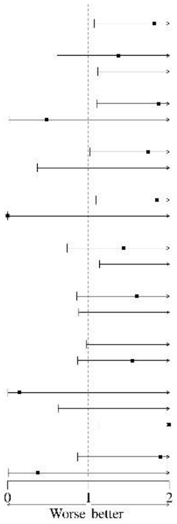

Supplementary table 7. Forest Map of VLDL Multifactor Analysis

Adjust for: age(smooth), height(smooth), weight(smooth), BMI(smooth) and age at menarche(smooth).

Variables	n (%)	OR (95%CI)	P	P for interaction
All patients	3491 (100.00)	0.94 (0.83 ~ 1.07)	0.364	
Overweight				0.039
No	1734 (49.67)	0.81 (0.67 ~ 0.99)	0.035	
Yes	1757 (50.33)	1.06 (0.90 ~ 1.25)	0.489	
Cardiovascular and cerebrovascular diseases				0.369
No	3309 (94.79)	0.95 (0.84 ~ 1.08)	0.437	
Yes	182 (5.21)	0.66 (0.30 ~ 1.47)	0.311	
DM				0.706
No	3325 (95.24)	0.94 (0.83 ~ 1.07)	0.334	
Yes	166 (4.76)	1.09 (0.52 ~ 2.26)	0.827	
CA				0.589
No	3445 (98.68)	0.94 (0.83 ~ 1.07)	0.353	
Yes	46 (1.32)	1.38 (0.35 ~ 5.53)	0.648	
History of abdominal surgery				0.147
No	2240 (64.16)	1.01 (0.86 ~ 1.18)	0.897	
Yes	1251 (35.84)	0.83 (0.67 ~ 1.03)	0.091	
History of cesarean section				0.154
No	2526 (72.36)	1.00 (0.86 ~ 1.15)	0.949	
Yes	965 (27.64)	0.81 (0.63 ~ 1.04)	0.094	
Menstrual regularity				0.077
No	808 (23.15)	1.22 (0.90 ~ 1.65)	0.209	
Yes	2683 (76.85)	0.90 (0.78 ~ 1.03)	0.123	
Amount of menses				0.416
Less	131 (3.75)	0.66 (0.30 ~ 1.44)	0.295	
More	377 (10.80)	1.11 (0.79 ~ 1.55)	0.544	
Normal	2983 (85.45)	0.93 (0.81 ~ 1.07)	0.340	
Dysmenorrhea				0.354
No	2986 (85.53)	1.04 (0.86 ~ 1.24)	0.702	
Yes	505 (14.47)	0.91 (0.74 ~ 1.12)	0.369	

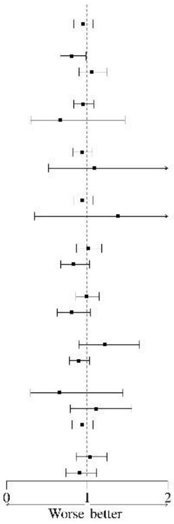

Supplementary table 8. Forest Chart for TC Single Factor Analysis

Adjust for: age(smooth), height(smooth), weight(smooth), BMI(smooth) and age at menarche(smooth).

Variables	n (%)	OR (95%CI)	P	P for interaction
All patients	3491 (100.00)	0.94 (0.83 ~ 1.06)	0.324	
Overweight				0.039
No	1734 (49.67)	0.82 (0.67 ~ 1.00)	0.048	
Yes	1757 (50.33)	1.05 (0.88 ~ 1.24)	0.606	
Cardiovascular and cerebrovascular diseases				0.367
No	3309 (94.79)	0.95 (0.83 ~ 1.08)	0.395	
Yes	182 (5.21)	0.65 (0.29 ~ 1.43)	0.281	
DM				0.696
No	3325 (95.24)	0.93 (0.82 ~ 1.06)	0.290	
Yes	166 (4.76)	1.07 (0.51 ~ 2.22)	0.859	
CA				0.699
No	3445 (98.68)	0.94 (0.83 ~ 1.07)	0.328	
Yes	46 (1.32)	0.00 (0.00 ~ Inf)	1.000	
History of abdominal surgery				0.208
No	2240 (64.16)	0.99 (0.85 ~ 1.16)	0.943	
Yes	1251 (35.84)	0.84 (0.67 ~ 1.04)	0.109	
History of cesarean section				0.246
No	2526 (72.36)	0.98 (0.85 ~ 1.13)	0.776	
Yes	965 (27.64)	0.81 (0.63 ~ 1.04)	0.095	
Menstrual regularity				0.066
No	808 (23.15)	1.28 (0.94 ~ 1.75)	0.118	
Yes	2683 (76.85)	0.89 (0.77 ~ 1.02)	0.090	
Amount of menses				0.494
Less	131 (3.75)	0.69 (0.29 ~ 1.65)	0.406	
More	377 (10.80)	1.10 (0.78 ~ 1.53)	0.593	
Normal	2983 (85.45)	0.93 (0.81 ~ 1.07)	0.303	
Dysmenorrhea				0.578
No	2986 (85.53)	1.03 (0.86 ~ 1.24)	0.735	
Yes	505 (14.47)	0.78 (0.34 ~ 1.82)	0.566	

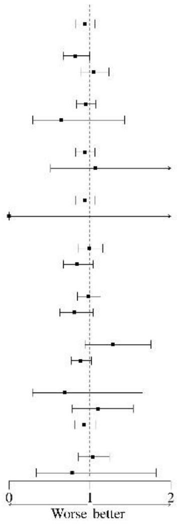

Supplementary table 9. Forest Map of TC Multifactor Analysis

Adjust for: age(smooth), height(smooth), weight(smooth), BMI(smooth) and age at menarche(smooth).

Variables	n (%)	OR (95%CI)	P	P for interaction
All patients	3491 (100.00)	1.21 (1.05 ~ 1.41)	0.010	
Overweight				0.225
No	1734 (49.67)	1.12 (0.92 ~ 1.38)	0.261	
Yes	1757 (50.33)	1.35 (1.08 ~ 1.70)	0.008	
Cardiovascular and cerebrovascular diseases				0.134
No	3309 (94.79)	1.19 (1.03 ~ 1.39)	0.020	
Yes	182 (5.21)	2.74 (0.98 ~ 7.65)	0.054	
DM				0.810
No	3325 (95.24)	1.22 (1.05 ~ 1.42)	0.010	
Yes	166 (4.76)	1.08 (0.41 ~ 2.88)	0.874	
CA				0.456
No	3445 (98.68)	1.22 (1.05 ~ 1.42)	0.009	
Yes	46 (1.32)	0.40 (0.01 ~ 11.86)	0.595	
History of abdominal surgery				0.033
No	2240 (64.16)	1.09 (0.90 ~ 1.32)	0.358	
Yes	1251 (35.84)	1.55 (1.20 ~ 2.01)	<.001	
History of cesarean section				0.297
No	2526 (72.36)	1.16 (0.98 ~ 1.38)	0.079	
Yes	965 (27.64)	1.40 (1.04 ~ 1.88)	0.026	
Menstrual regularity				0.138
No	808 (23.15)	1.03 (0.73 ~ 1.45)	0.863	
Yes	2683 (76.85)	1.33 (1.11 ~ 1.60)	0.002	
Amount of menses				0.337
Less	131 (3.75)	0.49 (0.09 ~ 2.58)	0.398	
More	377 (10.80)	1.50 (0.95 ~ 2.36)	0.080	
Normal	2983 (85.45)	1.20 (1.03 ~ 1.41)	0.023	
Dysmenorrhea				0.528
No	2986 (85.53)	1.17 (0.95 ~ 1.42)	0.134	
Yes	505 (14.47)	1.31 (0.97 ~ 1.76)	0.077	

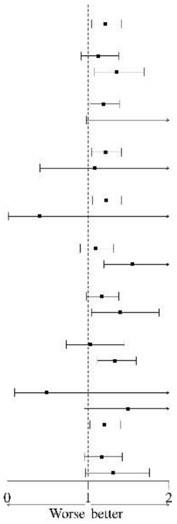

Supplementary table 10. Forest Chart for TG Single Factor Analysis

Adjust for: age(smooth), height(smooth), weight(smooth), BMI(smooth) and age at menarche(smooth).

Variables	n (%)	OR (95%CI)	P	P for interaction
All patients	3491 (100.00)	1.22 (1.05 ~ 1.42)	0.008	
Overweight				0.254
No	1734 (49.67)	1.15 (0.93 ~ 1.42)	0.209	
Yes	1757 (50.33)	1.37 (1.08 ~ 1.74)	0.009	
Cardiovascular and cerebrovascular diseases				0.159
No	3309 (94.79)	1.20 (1.03 ~ 1.40)	0.017	
Yes	182 (5.21)	2.68 (0.92 ~ 7.77)	0.069	
DM				0.748
No	3325 (95.24)	1.22 (1.05 ~ 1.42)	0.008	
Yes	166 (4.76)	0.98 (0.32 ~ 2.97)	0.972	
CA				0.381
No	3445 (98.68)	1.23 (1.06 ~ 1.43)	0.007	
Yes	46 (1.32)	0.00 (0.00 ~ Inf)	1.000	
History of abdominal surgery				0.028
No	2240 (64.16)	1.09 (0.90 ~ 1.32)	0.388	
Yes	1251 (35.84)	1.58 (1.22 ~ 2.06)	<.001	
History of cesarean section				0.219
No	2526 (72.36)	1.16 (0.98 ~ 1.38)	0.086	
Yes	965 (27.64)	1.42 (1.05 ~ 1.92)	0.021	
Menstrual regularity				0.185
No	808 (23.15)	1.07 (0.75 ~ 1.53)	0.715	
Yes	2683 (76.85)	1.32 (1.10 ~ 1.59)	0.002	
Amount of menses				0.387
Less	131 (3.75)	0.61 (0.10 ~ 3.65)	0.590	
More	377 (10.80)	1.47 (0.93 ~ 2.32)	0.101	
Normal	2983 (85.45)	1.21 (1.03 ~ 1.42)	0.017	
Dysmenorrhea				0.590
No	2986 (85.53)	1.17 (0.94 ~ 1.45)	0.152	
Yes	505 (14.47)	1.38 (0.28 ~ 6.73)	0.687	

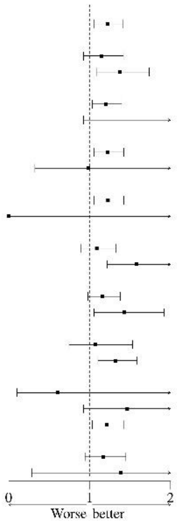

Supplementary table 11. Forest Map of TG Multivariate Analysis

Adjust for: age(smooth), height(smooth), weight(smooth), BMI(smooth) and age at menarche(smooth).

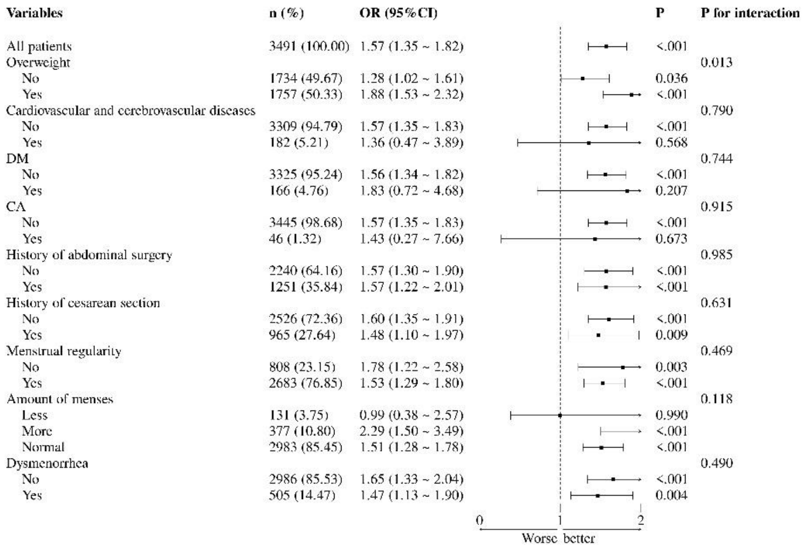

Supplementary table 12. Forest Chart for NHHR Single Factor Analysis

Adjust for: age(smooth), height(smooth), weight(smooth), BMI(smooth) and age at menarche(smooth).

Variables	n (%)	OR (95%CI)	P	P for interaction
All patients	3491 (100.00)	1.59 (1.36 ~ 1.85)	<.001	
Overweight				0.011
No	1734 (49.67)	1.25 (0.98 ~ 1.58)	0.068	
Yes	1757 (50.33)	1.91 (1.53 ~ 2.37)	<.001	
Cardiovascular and cerebrovascular diseases				0.825
No	3309 (94.79)	1.58 (1.36 ~ 1.85)	<.001	
Yes	182 (5.21)	1.42 (0.47 ~ 4.30)	0.537	
DM				0.826
No	3325 (95.24)	1.58 (1.35 ~ 1.84)	<.001	
Yes	166 (4.76)	1.87 (0.64 ~ 5.52)	0.256	
CA				0.899
No	3445 (98.68)	1.59 (1.36 ~ 1.85)	<.001	
Yes	46 (1.32)	7.72 (0.00 ~ Inf)	1.000	
History of abdominal surgery				0.983
No	2240 (64.16)	1.58 (1.31 ~ 1.92)	<.001	
Yes	1251 (35.84)	1.58 (1.23 ~ 2.03)	<.001	
History of cesarean section				0.734
No	2526 (72.36)	1.61 (1.35 ~ 1.93)	<.001	
Yes	965 (27.64)	1.50 (1.12 ~ 2.00)	0.007	
Menstrual regularity				0.417
No	808 (23.15)	1.88 (1.27 ~ 2.80)	0.002	
Yes	2683 (76.85)	1.53 (1.30 ~ 1.81)	<.001	
Amount of menses				0.113
Less	131 (3.75)	0.98 (0.34 ~ 2.79)	0.965	
More	377 (10.80)	2.30 (1.50 ~ 3.53)	<.001	
Normal	2983 (85.45)	1.53 (1.30 ~ 1.81)	<.001	
Dysmenorrhea				0.285
No	2986 (85.53)	1.70 (1.36 ~ 2.11)	<.001	
Yes	505 (14.47)	1.40 (0.44 ~ 4.45)	0.570	

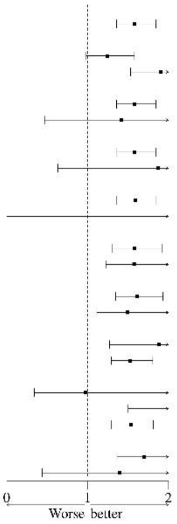

Supplementary table 13. Forest Map of NHHR Multivariate Analysis

Adjust for: age(smooth), height(smooth), weight(smooth), BMI(smooth) and age at menarche(smooth).
